# Development of Phenothiazine Hybrids with Potential Medicinal Interest: A Review

**DOI:** 10.3390/molecules27010276

**Published:** 2022-01-03

**Authors:** Marina C. Posso, Fernanda C. Domingues, Susana Ferreira, Samuel Silvestre

**Affiliations:** CICS-UBI-Health Sciences Research Centre, University of Beira Interior, 6200-506 Covilhã, Portugal; marina.posso@ubi.pt (M.C.P.); fcd@ubi.pt (F.C.D.); sms@ubi.pt (S.S.)

**Keywords:** phenothiazine, molecular hybridization, antimicrobial, antitumor, anti-Alzheimer, pharmacophore

## Abstract

The molecular hybridization approach has been used to develop compounds with improved efficacy by combining two or more pharmacophores of bioactive scaffolds. In this context, hybridization of various relevant pharmacophores with phenothiazine derivatives has resulted in pertinent compounds with diverse biological activities, interacting with specific or multiple targets. In fact, the development of new drugs or drug candidates based on phenothiazine system has been a promising approach due to the diverse activities associated with this tricyclic system, traditionally present in compounds with antipsychotic, antihistaminic and antimuscarinic effects. Actually, the pharmacological actions of phenothiazine hybrids include promising antibacterial, antifungal, anticancer, anti-inflammatory, antimalarial, analgesic and multi-drug resistance reversal properties. The present review summarizes the progress in the development of phenothiazine hybrids and their biological activity.

## 1. Introduction

In complex diseases there are many factors at play, such as genetic, lifestyle, and environmental factors that interact and lead to an observed phenotype [1]. In fact, diseases such as cancer, Alzheimer’s disease, microbial infections, obesity, and inflammatory diseases, among others, have complicated etiologies and pathogenesis [2]. As multiple factors are involved in these diseases, its cure or effective treatment frequently depends on achieving multiple targets and therefore, the use of a single drug with a single target may not be appropriate [3]. Although therapies with single drugs are available for some diseases, their efficacy can decrease due to several reasons, such as multidrug resistance [4]. Hence, the use of combined therapeutic approaches has been highlighted due to its ability to act on multiple targets and as a strategy to overpass the reduction of drug efficacy. This is based on the concomitant use of at least two different bioactive molecules with complementary pharmacological activity and different modes of action, which in turn can lead to enhanced therapeutic effects by different pathways, namely as a result of synergism [3]. However, these combinations of different drugs may also potentiate collateral effects and marked changes in their pharmacokinetic and pharmacodynamic profiles, which would influence their action [2]. Therefore, to find effective treatments for many diseases with reduced side-effects, researchers have been exploring the molecular hybridization approach [3].

**Figure 1:**
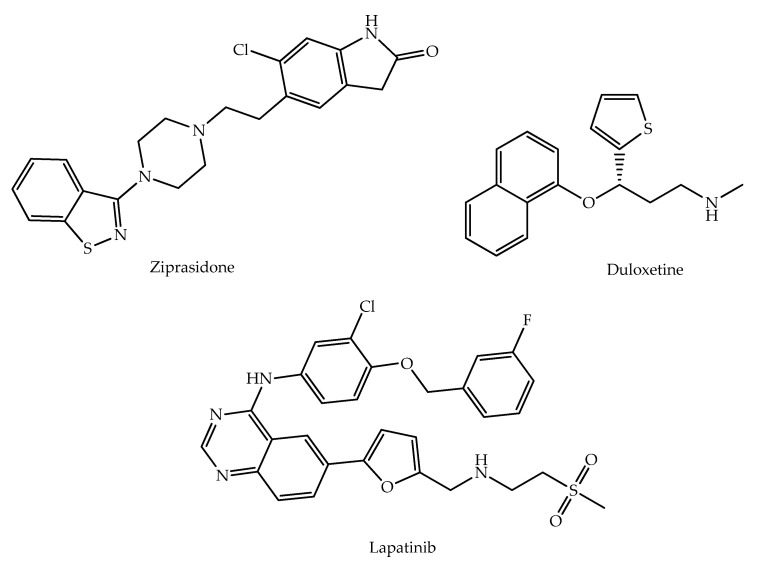
Three successfully marketed hybrid molecules.

One of the first examples of the application of molecular hybridization concept was the work of Fink et al., in 1996, who tried to combine acetylcholinesterase (AChE) and monoamine oxidase (MAO) inhibitory action in order to treat Alzheimer’s disease [5]. The molecular hybridization is based on coupling two or more pharmacophores, haptophores or entire drugs with different biological functions in one molecule using or not a linker chain [6]. These compounds should have additive or, preferentially, synergistic effects while bearing the expected pharmacological profiles for clinical use [7]. Hybrid molecules can also minimize the risk of drug-drug interactions and the potential development of drug resistance. In addition, this rational drug design strategy can allow for a reduction in dosage as well as in adverse drug reactions. Successfully marketed drug hybrids (relevant examples in Figure 1) include, among others, ziprasidone (antipsycothic), duloxetine (antidepressant), and lapatinib (used to treat breast cancer overexpressing ErbB2) [4].

**Figure 2:**
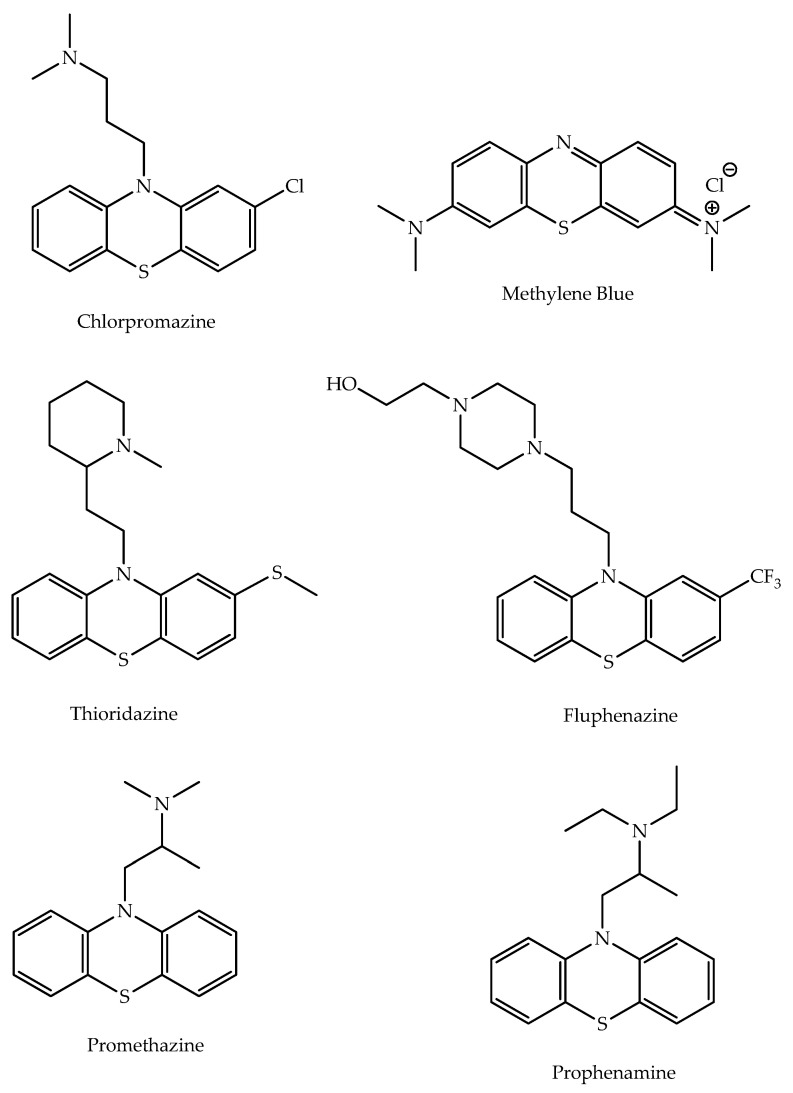
Examples of drug molecules containing phenothiazines scaffold: Methylene Blue, Chlorpromazine; Thioridazine, Fluphenazine, Promethazine, and Prophenamine [8].

Phenothiazines, a group of compounds characterized by the presence of the 10*H*-dibenzo-[*b*,*e*]-1,4-thiazine system (Figure 2), have been of high interest over the last several decades due to their wide medical applications [8]. The first synthesized compound bearing the phenothiazine skeleton was methylene blue (Figure 2). This dye was synthesized by Heinrich Caro in 1876 and was applied as an antimalarial among other medical conditions. Later, Heinrich August Bernthsen developed the synthesis of phenothiazine and defined its structure [9]. He obtained this product through heating diphenylamine and sulfur until the evolution of hydrogen sulfide ceased [10]. Although primarily used in clinics to treat schizophrenia, due to their antipsychotic effects, these compounds have also been of interest to treat nausea, methemoglobinemia, anxiety disorder, and acute intermittent porphyria. Phenothiazines exhibit diverse other biological activities, such as antibacterial, antiviral, antitumor, antiemetic, analgesic, antispasmodic, antihistaminic, anthelmintic, and redox activities [8,11,12]. This diversity of biological activities highlights the phenothiazine pharmacophore as a good candidate for molecular hybridization. Therefore, this scaffold has been selected for hybridization with other molecules to strengthen the effectiveness of the multitarget approaches or to obtain other potentially relevant bioactivities [4]. 

There are reports of different phenothiazine-based hybrids and different chemical approaches used in their development to enhance or modify their biological activities as well as to evaluate the effects of different substituents on their pharmacological activity. However, to the best of our knowledge, this review is the first to attempt a succinct compilation of the findings on phenothiazine hybrids. For this, an electronic search was performed in PubMed, ISI Web of Knowledge and other platforms using adequate keywords and their combinations, namely: “phenothiaz*” AND “hybrid*”; (Structure–activity relationship*) AND (phenothiaz* AND hybrid*), without temporal restriction. Therefore, in this review, we summarized the main phenothiazine hybrids published in the literature, from 2002 to 2021, as well as their therapeutic potential.

## 2. Anticancer Hybrids

Cancer represents a leading cause of death, being responsible for more than 9 million deaths and more than 19 million new cancer diagnoses were performed in 2020 [13]. Cancer develops as a consequence of genetic factors, and can be combined or not with external agents including physical, chemical, or biological carcinogens [14]. The result is the development of abnormal cells that can grow uncontrollably and spread to other parts of the organism. This uncontrolled cell growth is due to a lack of response to the appropriate signals regulating cell proliferation [15].

The available anticancer drug therapy presents relatively low efficacy and is associated with various side effects, some of which are limiting. In addition, there is always the risk of a fast development of drug resistance, which explains recurrences and relapses of cancer [16]. Thus, it is crucial to develop new chemotherapeutic agents displaying less toxicity and/or higher potency and cell selective effects, namely involving the design of multi-target agents. Therefore, hybrid drugs with multiple mechanisms of action can be of great value in cancer treatment. In this context, the phenothiazine system can be considered a good scaffold to develop new anticancer drugs, because some compounds of this class have potential for cancer treatment for many reasons, including for its binding affinity with calmodulin, by reverting resistance in drug resistant cell lines, by angiogenesis reduction and anti-invasive effects, by acting as an adjuvant in chemo- and radiotherapy due to its ability to enhance the radiation effect, for its antiproliferative and apoptosis induction in cancer cells, as well as its in vivo tumor suppressive activity, and suppression of autophagy-related proteins activating the via the PI3K/Akt/ mTOR/p70S6K signaling pathway [12,17,18,19]. Therefore, some of the most active hybrids with potential anticancer interest, based on their phenothiazine moiety, are discussed below.

**Figure 3:**
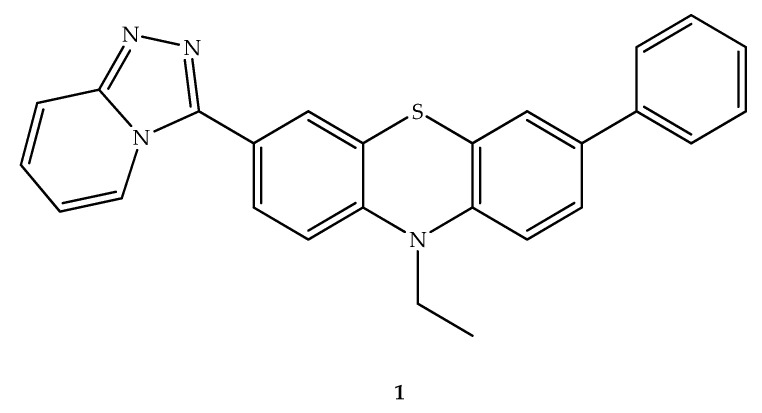
Chemical structure of 3-([1,2,4]triazolo[4,3-*a*]pyridin-3-yl)-10-ethyl-7-phenyl-10*H*-phenothiazine (**1**), an antitumor hybrid.

With a focus on the problem of numerous undesirable side effects of the conventional chemotherapeutic agents and the emergence of drug resistance, a new group of phenothiazine-triazolopyridine chemotherapeutic agents has been synthesized. The choice of these scaffolds was based on the fact that anticancer activity was demonstrated for trifluoperazine, which induced apoptosis in cancer cells and targeted multiple signaling pathways. Multidrug resistant (MDR) reversing effects were also demonstrated for the phenothiazine system [20]. In addition, the triazolopyridine moiety was also associated with antiproliferative activities against different human cancer cell lines. Within the group of compounds prepared, the [1,2,4]triazolo[4,3-*a*]pyridine bicyclic system was bound to a phenothiazine bearing a phenyl, originating a hybrid (compound **1**, Figure 3), which showed considerable apoptosis induction and cytotoxic effect against human breast cancer cell lines (MDA-MB-231, MDA-MB-468, MCF-7, SKBR3, and T47D), with the percentage of cell viability in the range of 6.2-31.5% at 100 µM. The determined half-maximum inhibitory concentration (IC_50_) of this new compound against the most affected cancer cell line, MCF-7, was 3.5-fold lower than the obtained inhibitory concentration in non-tumorigenic epithelial breast cells, MCF-10A, which and may indicate a selective effect and thus a reduced risk of side effects [21]. In this study, the structure-activity relationship (SAR) analysis demonstrated that the phenyl ring linked to the phenothiazine plays a role in cytotoxic activity. A molecular docking study of this hybrid compound supported the apoptosis induction effect, being possible to occur a binding to the cavity of tubulin, which can lead to the cell cycle disruption. Interestingly, the triazole ring can interact with the β-tubulin backbone NH of the T276 residue, which also occurs with the oxetane ring of anti-mitotic chemotherapeutic paclitaxel [21,22]. In vitro studies seemed to be in agreement with in silico results, which demonstrated a cell cycle arrest in G2/M and induced apoptosis [21].

**Figure 4:**
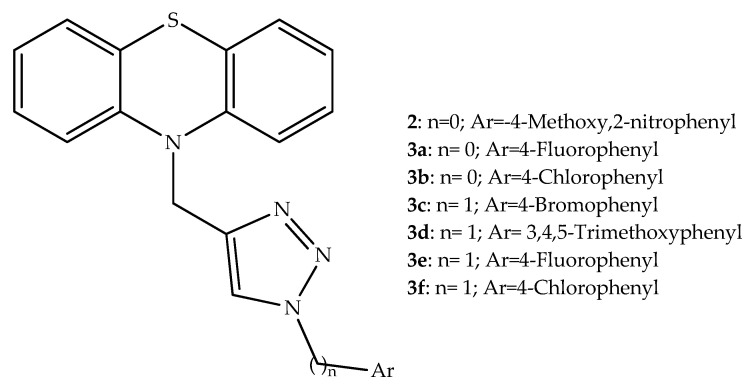
Chemical structures of the most active phenothiazine-1,2,3 triazole hybrids with antiproliferative activity.

Microtubules are hollow tubes made of close, tightly linked molecules known as tubulin heterodimers. Microtubules play a crucial role in many essential functions within the cell, as well as being important in cell division, and interference in this system can lead to cellular death. Several anticancer drugs target microtubules dynamics, leading to anticancer effects. Four tubulin binding sites were involved in the effects of most of the antimitotic drugs currently available for clinical use or in development. One of those sites is the colchicine binding place, which promotes microtubule depolymerization [23]. Liu et al. (2019) applied a molecular hybridization approach to develop new antitumor agents where a 1,2,3-triazole unit, which is associated with a tubulin polymerization inhibitory effect, was linked with a phenothiazine scaffold. Among the compounds synthetized in this series, hybrid **2** (Figure 4) showed to be the most potent antiproliferative agent against the studied gastric cancer (MGC-803, MKN28 and MKN45) cell lines. This active product was also found to have a good selectivity in relation to normal cells (GES-1). In addition, the antiproliferation ability of compound **2** in MGC-803 cells was further explored and it was demonstrated to be dependent on time of exposition and hybrid concentration. Moreover, this phenothiazine derivative led to the inhibition of MGC-803 cells migration in a concentration-dependent way by regulating the wnt/β-catenin signaling pathway and can influence the epithelial-mesenchymal transition process through upregulating the expression level of E-cadherin and downregulating *N*-cadherin, vimentin, and active-MMP2. Furthermore, compound **2** in vitro inhibited the tubulin polymerization and through in vivo studies it was demonstrated that this phenothiazine derivative effectively inhibited the MGC-803 xenograft tumor growth in severe combined immunodeficiency mice. The SAR analysis of compound **2** indicated that the substituent on the phenyl ring affected the observed inhibition [24].

Other 1,2,3-triazole-based hybrids were developed by Ma et al. (2017) by linking this unit to a phenothiazine through a methylene bridge, similarly to compound **2**. These synthesized hybrids were tested for antiproliferative activity against stomach (MGC-803), oesophageal (EC-109), prostatic (PC-3), breast (MCF-7) and hepatocellular (HepG-2) cancer cell lines, and all showed antiproliferative activity. Among this group of compounds, the derivatives **3a** and **3b** (Figure 4) presented a more potent antiproliferative effect (IC_50_ range 0.5–9.6 µM) than the determined effect for the standard drug that is used, 5-fluorouracil (IC_50_ range 10.7–17.6 µM). Considering the biological data obtained, a bulky, hydrophobic, and electron-withdrawing substituent at the phenyl ring bound to the 1,2,3-triazole unit may play an essential role in the antiproliferative activity, since compound **3b**, bearing a *p*-Cl atom, was the most potent against all the selected cell lines [25].

Further studies performed by the same research group involved the preparation of similar phenothiazine-1,2,3-triazole hybrids (compounds **3c**–**f**, Figure 4), again by the already described click chemistry approach. This study brought more insights about the SAR within this class of compounds. The authors demonstrated that the substituents in the phenyl ring bound to 1,2,3-triazole also displayed a relevant effect on the antiproliferative activity against breast cell cancer lines. In fact, the replacement of the electron-withdrawing groups, such as F, Cl and Br, (**3e**, **3f** and **3c**), by an electron-donating group (a methyl) led to an increase of the antiproliferative activity against all cancer cell lines. The best antiproliferative effects were observed for the hybrid with a 3,4,5-trimethoxy substituent on the phenyl ring (**3d**), specifically for breast cancer cell lines. Furthermore, the results for this derivative were even better than those determined for the control drug, 5-fluorouracil. In MCF-7 cells, compound **3f** led to morphological changes, including the formation of apoptotic bodies, and to an increase in the percentage of apoptotic cells. Moreover, the expression of proteins involved in the apoptosis was evaluated by Western blot and an increase in the expression of Bad, Bax, and DR5 and a decreased expression of Bcl-2 and Parp were observed [26].

**Figure 5:**
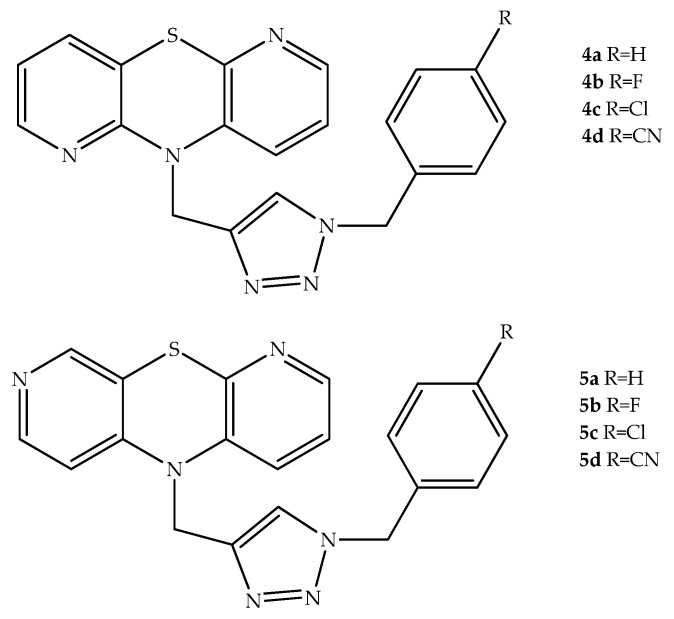
Chemical structures of 1,2,3-triazole and diazaphenothiazine hybrids with antiproliferative effects.

Morak-Młodawska et al. (2019) also synthesized a series of triazole-substituted diazaphenothiazine hybrids (e.g., compounds **4a**–**d**; **5a**–**d**) and evaluated their antitumor action against SNB-19 (glioblastoma), Caco-2 (colorectal carcinoma), MDA-MB231 (human breast cancer), and A549 (lung cancer) cells, while using normal human dermal fibroblasts (NHDF) for selectivity studies. Within the group of compounds prepared and evaluated, the most active against all cancer cells was **4c**, bearing a *p*-chlorobenzyl group bound to the 1,2,3-triazole ring, which presented better IC_50_ values than cisplatin, a platinum-based chemotherapy drug used in cancer treatment (Figure 5). Other analogues also showed similar antiproliferative activity against all cancer cell lines. However, within the group of the most potent antiproliferative compounds, only the derivative **4c** clearly presented low cytotoxicity to NHDF. Given this, the authors considered that compound **4c** had the highest potential and further explored its possible mechanism of anticancer activity. Interestingly, this hybrid clearly reduced the expression of pro-survival gene Bcl-2 in SNB-19, A549, and MDA-MB231 cell lines, and increased the expression of pro-apoptotic Bax and cyclin-dependent kinase inhibitor *CDKN1A* in all cancer cells, which may indicate that compound **4c** can have apoptosis-inducing effects [27].

**Figure 6:**
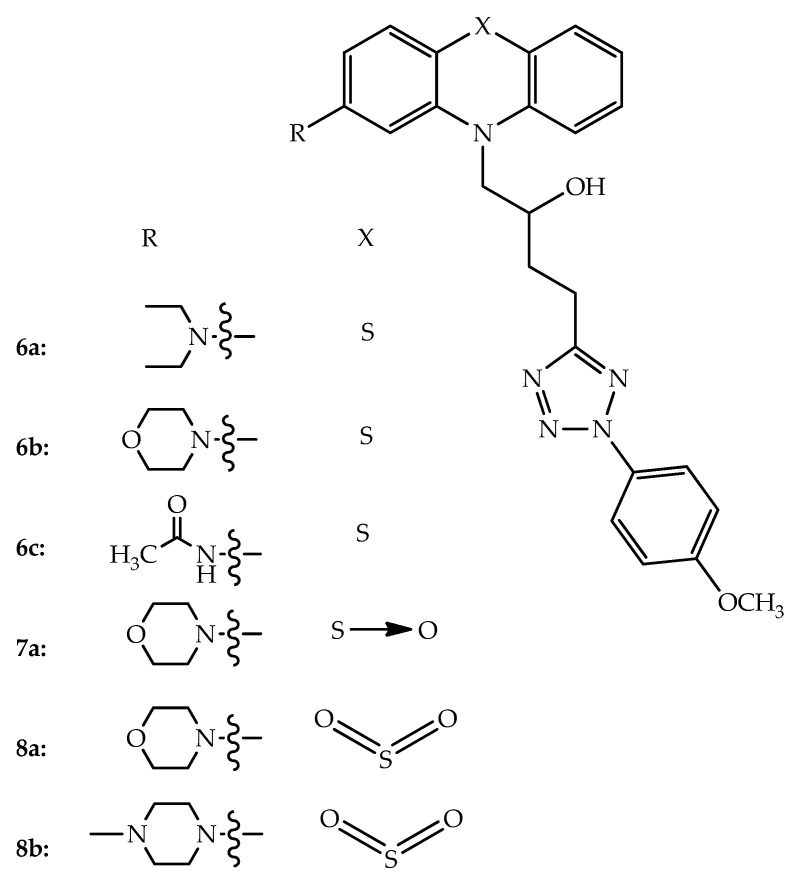
Chemical structures of *N*-(2-hydroxybutyl)-2-aminophenothiazines bound to 4-methoxyphenyltetrazole with P-glycoprotein inhibitory activity.

P-glycoprotein is an ATP-binding cassette (ABC) transporter involved in drug resistance in cancer cells. To develop new anticancer therapeutics against multidrug resistant tumors, compounds bearing phenothiazine and secondary amino moieties (Figure 6) with high antitumor interest and inhibitory properties in MDR cancer cells were prepared. Specifically, the authors synthesized *N*-tetrazolylbutylphenothiazines with different amines at position 2 of the phenothiazine tricyclic system and, by oxidation, some sulfoxide and sulfone hybrids were also obtained. Interestingly, when testing their inhibitory properties of the ABC transporter P-glycoprotein (ABCB1) in primary rat hepatocytes, the compounds with the 4-methoxyphenyltetrazole moiety (**6a**, **6b** and **6c**) showed the highest efficacy, comparable to verapamil, which is a known P-glycoprotein inhibitor. Sulfur atom oxidation (**7a**, **8a** and **8b**) significantly enhanced the potential of these compounds as P-glycoprotein inhibitors, with the sulfone derivative **8a** exhibiting the greatest potency. Further, the new compounds were nontoxic to hepatocytes [28]. The antiproliferative activity of similar hybrids developed by the same research group, showed compounds **6c**, **7a** and **8a** as the most active against multidrug-resistant COLO 320 colon adenocarcinoma cells, presenting antiproliferative and potent cytotoxic activity. These phenothiazine derivatives also have shown synergism with doxorubicin against MDR colonic adenocarcinoma cells. The sulfone **8b**, which was prepared by the sulfur oxidation of the most active derivative found in this study, was the only compound that also led to apoptosis induction [29].

**Figure 7:**
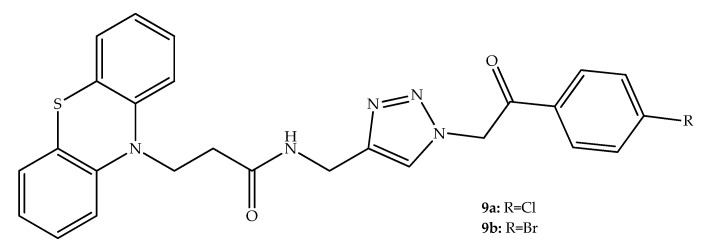
Chemical structures of hybrids bearing the phenothiazine core and *N*-acylated 1,2,3-triazole with antitumor interest.

Farnesyltransferase (FTase) can also be seen as a potential target for cancer treatment approaches. The FTase enzyme catalyzes the addition of a lipid group to the terminal carboxyl of several proteins, including the Ras protein, thus exhibiting a protooncogenic role [30]. FTase inhibitors can also have alternative uses, and it is important to mention that the first inhibitor was approved in November 2020 for Hutchinson-Gilford progeria syndrome [31]. In this context, Belei et al. (2012) discovered that hybrids bearing a phenothiazine and 1,2,3 triazole in their structure have FTase inhibitory properties. These authors prepared three new series of compounds with these structural characteristics, and it was demonstrated that the FTase inhibitory potency is within the low micromolar range for several of these phenothiazines. Considering the SAR data analysis, the authors concluded that *p*-chloro and *p*-bromo substitutions on the phenyl ring improved the compounds FTase inhibitory activity. In addition, a shorter length of the carbon chain between the phenothiazine nitrogen and the amide group led to a decrease in the FTase inhibition. Several hybrids with FTase inhibitory properties were selected for cytotoxicity studies using cancer cell lines. Among the tested compounds, the hybrid **9a** (Figure 7) exhibited the most relevant antiproliferative activity (68% inhibition of leukemia HL-60 (TB), 40% inhibition of leukemia MOLT-4, and 38% inhibition of prostate cancer PC-3 cell lines at 10 µM). It was also interesting to observe that compound **9b**, which has relevant FTase inhibitory effects, led to a relatively similar reduction of proliferation on most of the cancer cell lines. These results evidenced that the combination of phenothiazine and 1,2,3 triazole groups can be interesting in the development of new FTase inhibitors [32].

**Figure 8:**
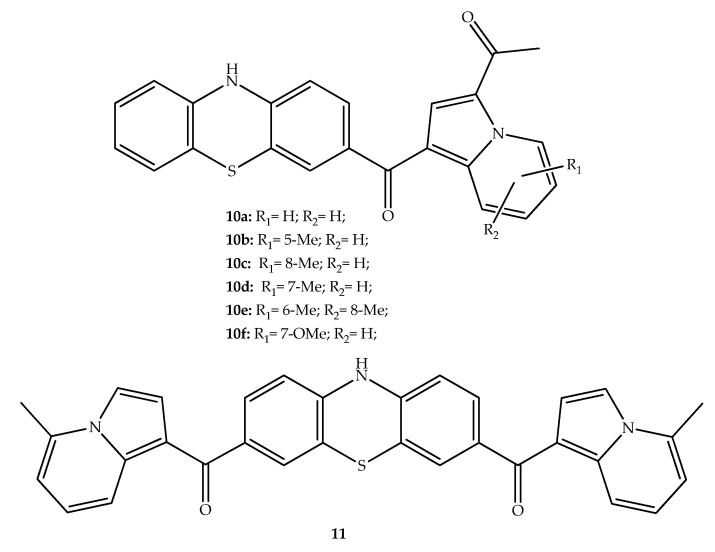
Chemical structures of phenothiazine-indolizine derivatives with antiproliferative activity.

Moise et al. (2020) aimed to create new FTase and tubulin polymerization inhibitors with antitumor interest by hybridizing indolizines with the phenothiazine tricyclic system. The *N*-acylation of the phenothiazine ring did not result in a remarkable antimitotic activity. However, the synthesized phenothiazine-ketones **10a**, **10b**, **10c** and **10d** (Figure 8) were presented as relevant tubulin polymerization inhibitors. In addition, the ketone hybrids **10a**–**f** and **11** also inhibited FTase. A screening in 60 human tumor cell lines revealed that the phenothiazine-ketone **10a** displayed the most significant antitumor effect, inhibiting cell proliferation at low nanomolar concentrations on all cancer cells (half-maximum growth inhibition (GI_50_): 1.8–6.5 nM). The phenothiazine system was unsubstituted on the nitrogen atom and the presence of indolizine bearing methyl or methoxy substituents are important for this activity. The authors highlighted that even though compound **10a** is not the best dual inhibitor of tubulin polymerization and FTase, its potent effect in cancer cells lines suggests that this hybrid can more effectively penetrate the cell membrane, eventually reaching other biological targets [33].

**Figure 9:**
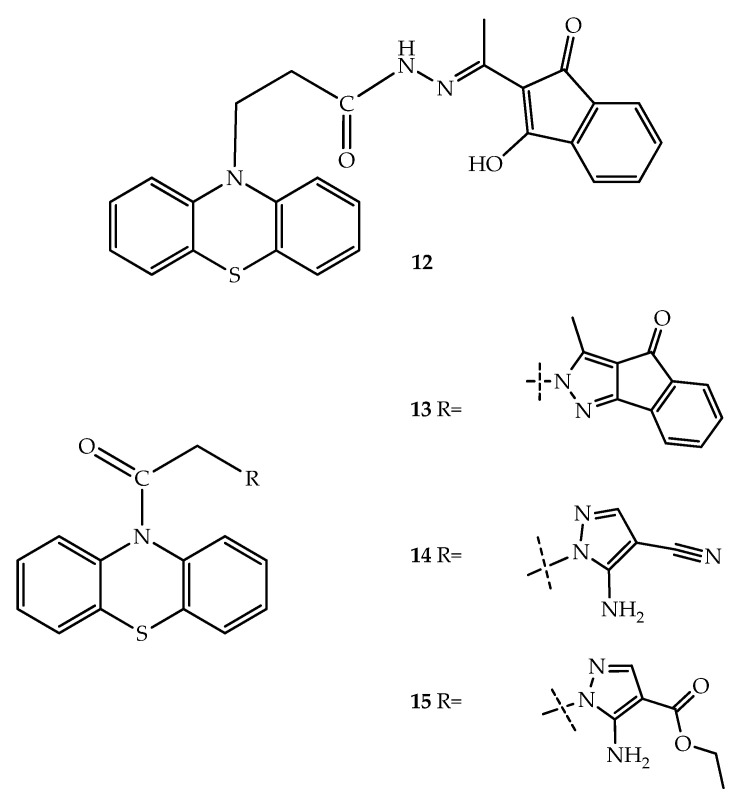
Chemical structures of the most active pyrazolo-phenothiazine and similar compounds with FTase inhibitor activity.

In order to develop hybrid molecules using the FTase protein as a target, Baciu-Atudosie et al. (2012) synthesized a series of phenothiazine derivatives, and several of these incorporated a pyrazole ring. The screening for FTase inhibitory activity revealed that the three most active compounds were **12**, **13**, **14**, and **15** (Figure 9), inhibiting near 95.5%, 67.7%, and 94.9% of the activity at 100 µM, respectively. Among these, compound **13** at 10 µM inhibited the proliferation of 57 cancer cell lines (9.6–55.7%), including leukemia, central nervous system (CNS), colon, non-small cell lung, melanoma, renal, ovarian, prostate, and breast cancer cell lines. The highest activity was observed for compound **13** against colon (HCT-166) and melanoma (LOX IMVI, SK-MEL-5) cancer cell lines, with values higher than 50% of growth inhibition at 10 µM. In addition, at the same concentration, compound **15** also lead to 83.2% growth inhibition of leukemia HL-60 (TB) cells [34].

**Figure 10:**
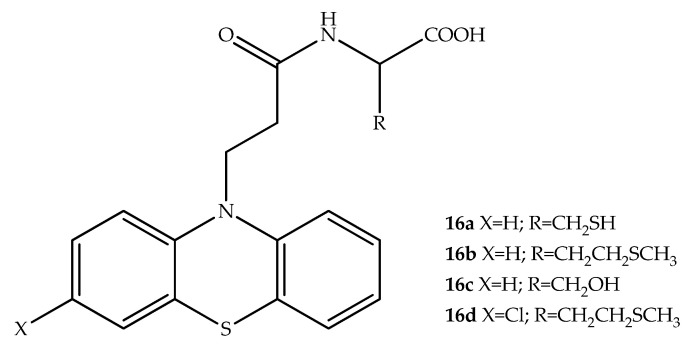
Chemical structures of amino acid-phenothiazine with FTase inhibitory activity. The most promising FTase inhibitor was **16d**.

As FTase’s recognize, modifiy and activate cysteine residues at the carbonyl terminus of the tetrapeptide (CaaX) and catalyze the covalent link of a farnesyl group [35], Dumitriu et al. (2015) designed CaaX competitive inhibitors, by modification of the phenothiazine scaffold by introducing aminoacids, to synthesize zinc chelating compounds. These phenothiazine derivatives were prepared by combination with cysteine, methionine, serine, and valine residues. Among the hybrids synthesized, the carboxylic acids were found to have relevant in vitro activity, supporting the notion that the carboxyl group has a higher zinc chelating capacity, acting as a haptophore group. The strongest FTase inhibition was achieved by compounds **16a**, **16b**, **16c** and **16d** (Figure 10) [36]. Docking studies revealed that the phenothiazine derivative **16a** should be placed on the same side of the protein active site and is oriented in the same direction of the derivative **16d**. The 2-chlorophenothiazine derivative **16d** showed the highest FTase inhibition, being determined the lowest IC_50_ value for this compound. Quantitative SAR studies show that the phenothiazine skeleton plays an important role in the human farnesyltransferase inhibition. Therefore, FTase inhibitory properties are critically influenced by carboxylic acid locations near zinc atoms of proteins, which is an interaction important for the inhibitory properties of the enzyme [36].

**Figure 11:**
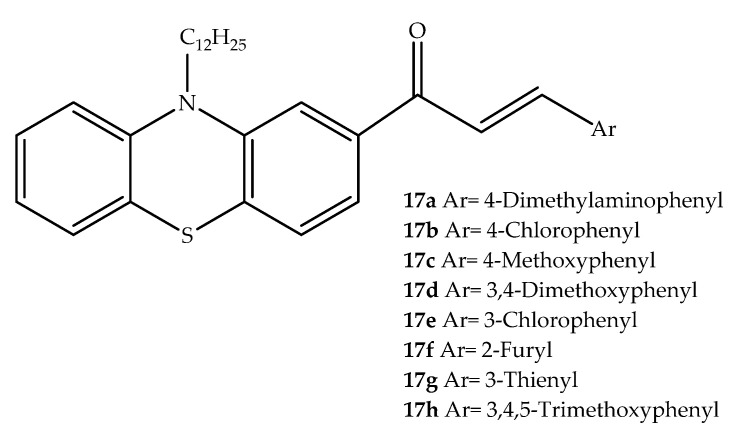
Chemical structures of chalcone-phenothiazine hybrids with antitumor interest.

Al Zahrani et al. (2020) synthesized various hybrids by linking the phenothiazine system to a chalcone (Figure 11) and studied their cytotoxicity *in vitro*. In the tested human hepatocellular carcinoma (HepG2) and breast cancer (MCF-7) cell lines, an IC_50_ range of 7.14–13.0 µM was determined for compounds **17b** and **17h**. In contrast, the hybrid **17e**, a chlorine-containing isomer of **17b**, was shown to be the least effective compound against the tested cell lines. Therefore, a relationship between group position in chalcone aryl rings and cytotoxicity appears to exist. Interestingly, compounds **17a**–**d** and **17f**–**h** (Figure 11) displayed a radical-scavenging activity similar to ascorbic acid, but lower than gallic acid. The planar structure of the phenothiazine core bound to the chalcone system can explain the radical stability as well as the anticancer effects [37].

**Figure 12:**
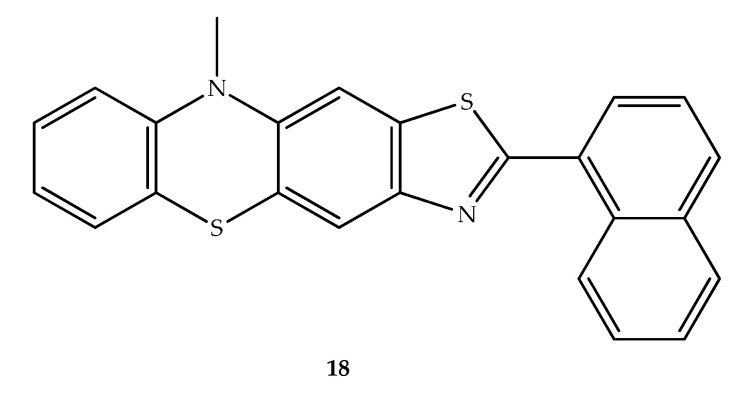
Chemical structure of the most potent antiproliferative compound synthesized by Brem et al. (2017): 2-(naphthalen-1-yl)-10-methyl-10*H*-thiazolo[5,4-*b*]-phenothiazine [38].

Due to the diverse biological properties of phenothiazines and benzothiazoles, including potential anticancer effects, Brem et al. (2017) designed substituted-thiazolo[5,4-*b*]phenothiazines as potential antileukemic agents. Most of these hybrids showed a relevant antiproliferative activity on human promyelocytic and monocytic leukemia cell lines (HL60 and THP-1) and no significant effect in the growth of normal peripheral blood mononuclear cells (PBMC) in high concentration was observed. Of these, the most active was compound **18** (Figure 12) against THP-1, with a cytotoxic effect comparable to cytarabine, a clinically used antineoplastic agent. The compound displayed no cytotoxicity in normal PBMC cells. On both THP-1 and HL-60 cancer cell lines, the hybrid **18** affected the metabolic activity and induced apoptosis. The presence of the thiazole ring linked with phenothiazine increased the biological activity when compared with *N*-(10-methyl-10*H*-phenothiazin-3-yl)naphthalene-1-carbothioamide [38].

**Figure 13:**
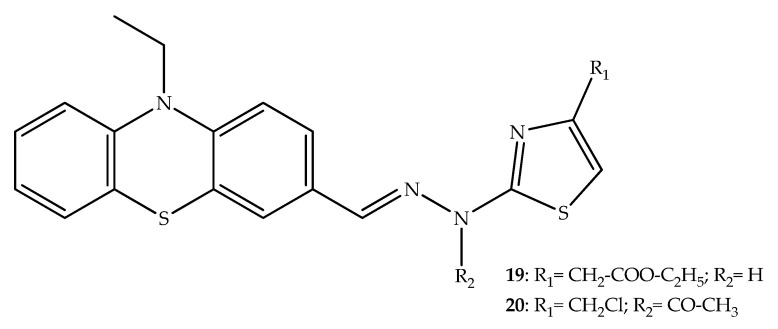
Chemical structure of the most active phenothiazinyl-thiazolyl-hydrazine derivatives with cytotoxic effects.

To shorten the reaction times and increase yields, Ignat et al. (2012) synthesized phenothiazinyl-thiazolyl-hydrazines using microwave irradiation. Afterward, the compounds were tested against colon carcinoma (CC531S) and hepatic tumor (HepG2) cells. The antiproliferative activity of the most active compounds **19** and **20** (Figure 13) were near to the observed for cisplatin. The cytotoxic activity was potentiated by the chloromethyl in position 4 of the thiazole ring [39]. This activity may be related to the phenothiazine pharmacophore, as observed by the cytotoxicity displayed by thioridazine, which possesses this tricyclic system, in HepG2 cells through a Ca^2+^ independent manner at the 30–80 µM concentration [40].

**Figure 14:**
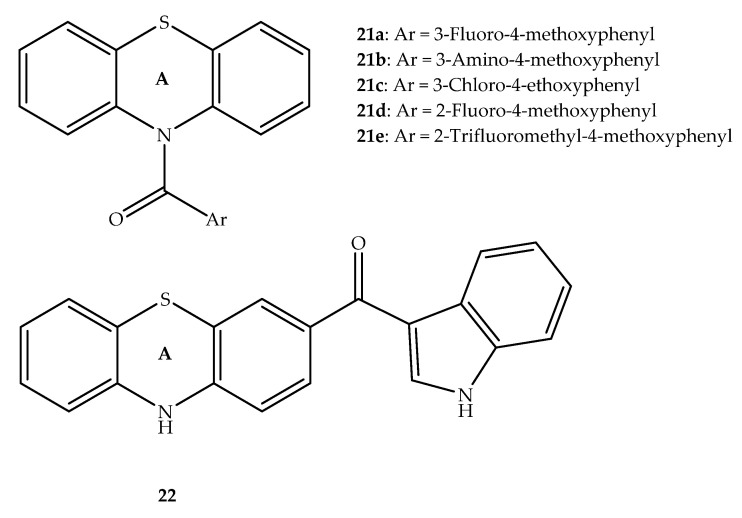
Chemical structure of phenothiazine-based hybrids linked with analogs of phenstatin [42,46].

Combretastatin A4 is a bioactive stilbene with two rings: a trimethoxy A ring and an *m*-hydroxi and *p*-methoxy B ring linked by an ethene bridge. This molecule is similar to phenstatin, in that the two functionalized rings that are similar to combretastatin A4 are bound by a stable ketone group [41]. Considering the potential of combretastatin A4 to bind the colchicine site and act as a tubulin polymerization inhibitor, Abuhaie et al. (2013) linked phenothiazine and the phenstatin ring A to develop new antitumor hybrid compounds (Figure 14). The derivatives **21a** and **21b** showed a good anti-tubulin polymerization action. Further, the hybrid **21b** had the most potentin vitro antiproliferative activity against leukemia (K-562 and SR), non-small cell lung (NCI-H522), colon (HCT-15, KM12 and SW-620), melanoma (M14, MDA-MB-435, and SK-MEL-5), ovarian (NCI/ADR-RES), CNS (SF-295), and breast (MCF-7, and MDA-MB-468) cancer cell lines, with GI_50_ values in the nanomolar range. The phenothiazine derivative **21c**, bearing a 3-chloro-4-ethoxy phenyl, also had a cancer cell growth inhibition comparable to the fluoro-derivative. The results obtained from in vitro cytotoxicity studies revealed that product **22** also had a moderate cytotoxic effect against cancer cell lines and tubulin polymerization when compared with **21a** and **21b**. These bioactive compounds showed the ability to bind to the colchicine binding site of tubulin, as showed by the docking analysis using the crystallographic structure of a heterodimer of α and β-tubulin, and the binding site co-crystallized with thiocolchicine [42]. This result is congruent with the activity of other *N*-heterocyclic hybrids that possess antiproliferative activity and inhibit tubulin polymerization [43,44,45].

Other phenstatin analogues were developed by the same research group involving the combination of phenyl, methoxyphenyl or 1*H*-indol-3-yl with the phenothiazine scaffold which acts as the phenstatin B ring. Several structural changes on its classical 3′-hydroxy-4′-methoxyphenyl B ring were explored and these modifications have been shown to also allow a relevant tubulin polymerization inhibition [46]. Among the phenothiazine-amides tested by Ghinet et al. (2016), the most potent tubulin polymerization inhibitors (Figure 14) were the derivatives bearing 3′-fluoro-4′-methoxyphenyl (**21a**), 3′-amino-4′-methoxyphenyl (**21b**), 2′-fluoro-4′-methoxyphenyl (**21d**) and 2′-trifluoromethyl-4′-methoxyphenyl (**21e**) moieties [46]. In this study, eleven synthesized derivatives were tested for their antiproliferative effects against 60 cancer cell lines, including several multidrug-resistant tumor cell lines, with all hybrids exhibiting cytotoxic effects against at least one cell line. The most pronounced cell growth inhibitory effect was found in human melanoma-adenocarcinoma MDA-MB-435 cells for compound **21a**. Also, compound **21e** showed a marked effect on human acute lymphoblastic leukemia CCRF-CEM cells [46]. Therefore, this study revealed that the phenothiazine phenstatin hybrids might be potential antineoplastic agents due to their ability to inhibit tubulin polymerization, which is similar to the results obtained by Abuhaie et al. (2013) [42,46].

**Figure 15:**
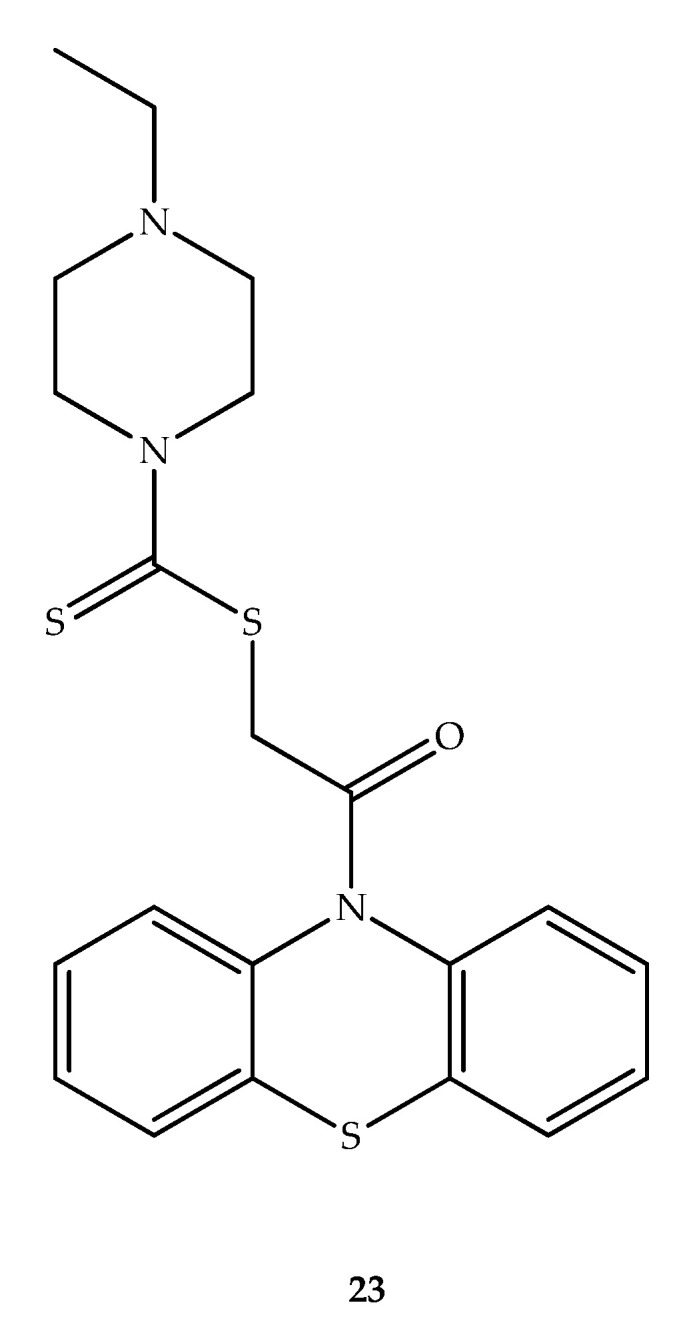
Chemical structure of phenothiazine-dithiocarbamate (**23**) hybrid with potent anticancer activity.

In the design of new anticancer cell cycle blockers, Fu et al. (2017) applied a molecular hybridization method using phenothiazine and dithiocarbamate scaffolds [47]. Phenothiazine and dithiocarbamate derivatives exhibited weak growth inhibition of three cancer cell lines (PC-3, EC-109 MGC-803). However, the *N*-alkylated derivative (**23**) has shown a good antiproliferative effect against the tested cancer cells. Therefore, for cell growth inhibitory activity, the ethyl group on the piperazine moiety played an important role. Within the group of compounds prepared, compound **23** (Figure 15) was shown to inhibit PC-3 cell growth, with an IC_50_ of 11.59 μM. Despite not inducing apoptosis in these cells, compound **23** dose-dependently interrupted the cell cycle in the G1 phase through negative regulation of cyclin D1 and the CDK4 complex [47]. As this complex is associated with the transition to the S phase of the cell cycle, there is a simultaneous decrease in the number of cells in the G2 and S phases. Therefore, the authors suggested this compound as a potential new cell cycle blocker [47]. The activity of these derivatives can be compared to the antiproliferative and apoptosis-inducing activity of thioridazine through the Wnt/β-catenin signaling pathway [48].

**Figure 16:**
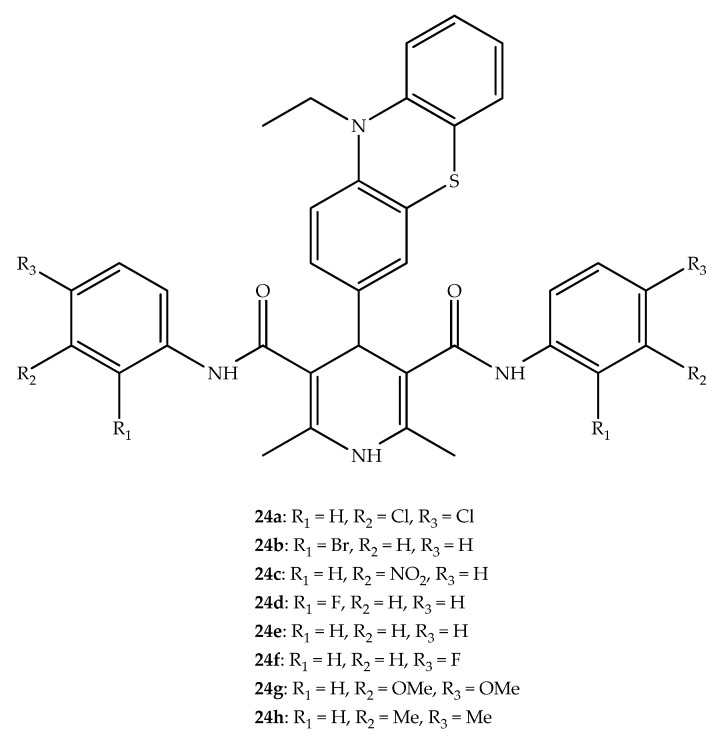
Chemical structure of the most potent phenothiazinyldihydropyridine dicarboxamides hybrids as antiproliferative agents.

Phenothiazinyldihydropyridine dicarboxamides (Figure 16) were synthesized and their cytotoxicity was evaluated against pancreatic cancer cell lines SW1990, AsPC1, BxPC3, and Panc1, as well as in non-cancerous cells MRC5. The presence of *m*- and *p*-chloro atoms in the aryl ring of the amide structural motif (**24a**) enhanced the activity of these compounds against all human pancreatic cancer cell lines. An analysis of the radical scavenging activity by a DPPH assay showed that bromo and fluoro atoms in *ortho* position or an *m*-nitro on the aryl ring influences this antioxidant effect. In fact, the hybrids **24b**, **24c** and **24d** act as antioxidants with an activity comparable to the standard, i.e., ascorbic acid [49].

**Figure 17:**
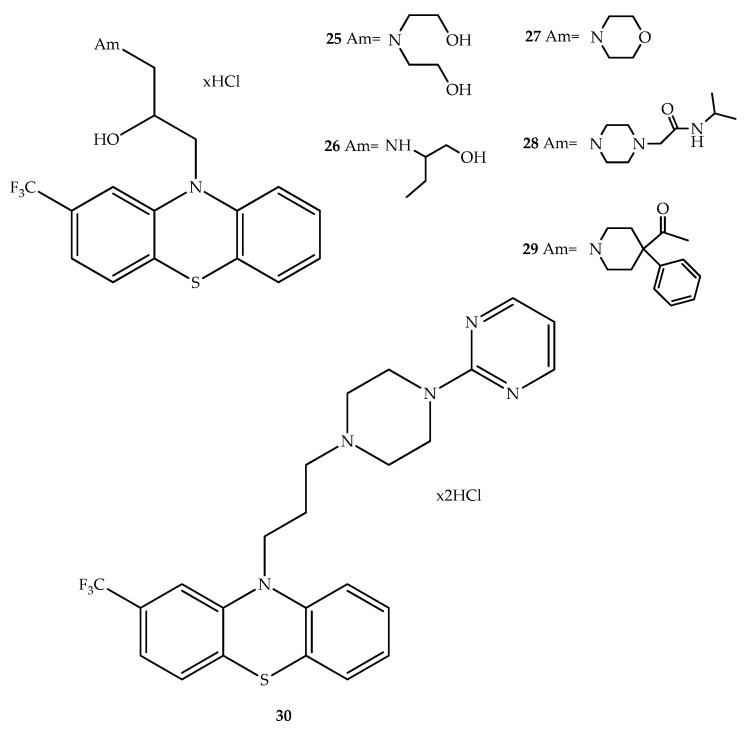
Chemical structure of the most potent phenothiazinyldihydropyridine hybrids as antitumor compounds.

Fluphenazine analogues were synthesized and evaluated for their apoptotic activity and cytotoxicity in human lymphocytes which were genotoxically damaged in vitro with benzo[*a*]pyrene. Compounds **25–30** (Figure 17) exerted a pro-apoptotic effect stronger than that of fluphenazine and compounds **25**, **28**, **29** and **30** exhibited the weakest cytotoxic effect on normal lymphocytes isolated from venous blood from five health donors, showing selectivity to cancer cells [50].

**Figure 18:**
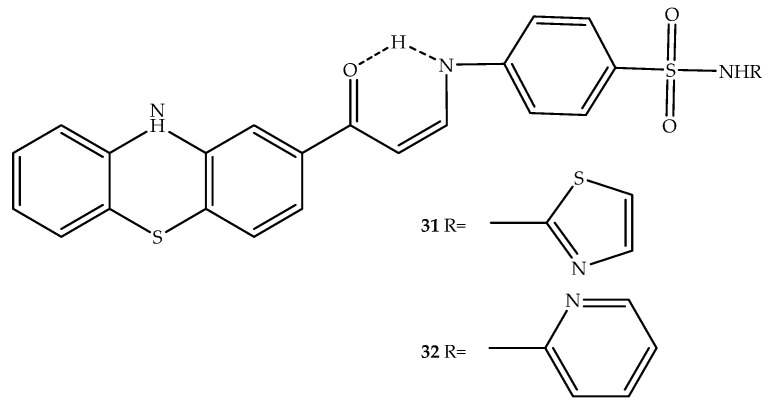
Chemical structure of the most potent hybrids with sulfonamide moiety with anticancer activity.

Novel phenothiazine/sulfonamide hybrids were developed by Ghorab et al. (2017), with compounds **31** and **32** (Figure 18) being more effective than doxorubicin against human breast cancer cells (T47D), which is a cancer cell line that possess receptors for a variety of steroids [51]. To determine how these compounds exert their anticancer properties, their ability to inhibit aromatase and thus block the biosynthesis of estrogens, hormones that stimulate hormone responsive breast cancer and metastization, was also tested. Interestingly, compound **31** was able to inhibit aromatase by 68.3% at 100 μM and the IC_50_ was 5.67 µM, while letrozole, a clinically used nonsteroidal aromatase inhibitor showed 58% inhibition and IC_50 (aromatase)_ = 29.5 µM. The most potent aromatase inhibitors were compounds **31** and **32** having the lowest IC_50_ values on the target enzyme (5.67 µM and 6.7 µM, respectively). Additionally, compound **31** displayed an apoptotic effect by up-regulating Bax expression levels, down-regulating antiapoptotic Bcl2, and activating the proteolytic caspase cascade. In docking studies, it was observed that the phenothiazine ring fits into the hydrophobic pocket of the aromatase binding site in a similar way to androstenedione, the co-crystallized ligand [51].

**Figure 19:**
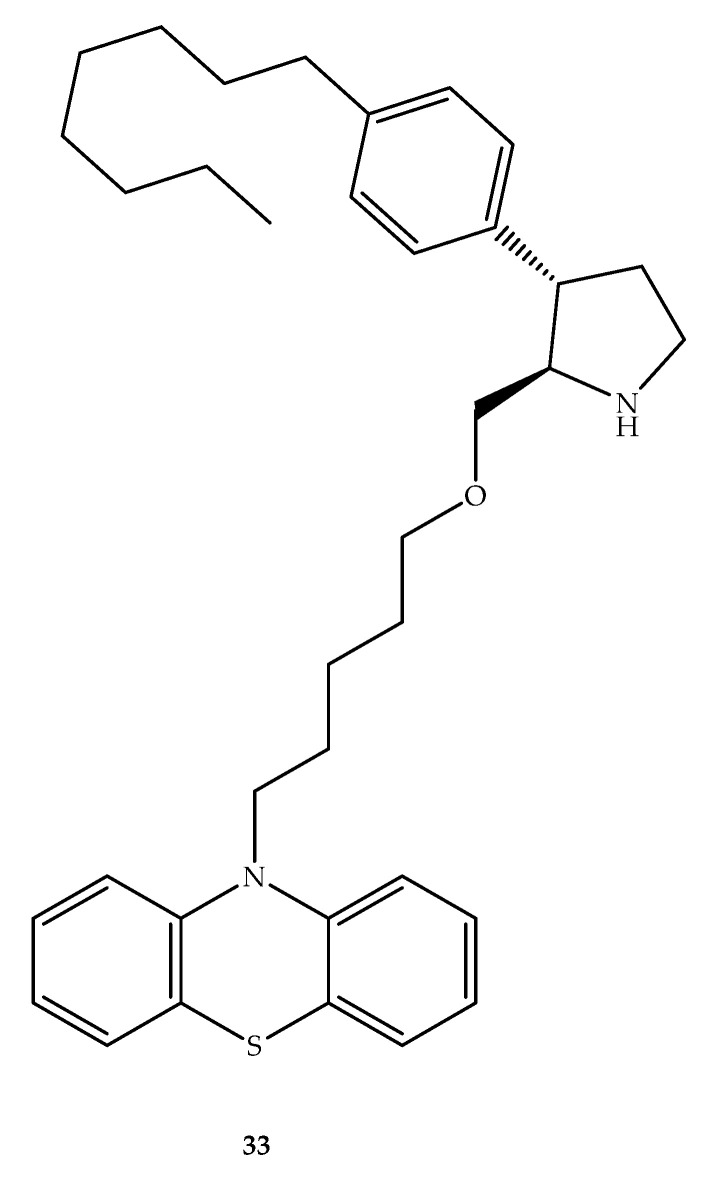
The chemical structure of phenothiazine-sphingolipid hybrid **33**.

Garsi et al. (2019) combined phenothiazine or phenoxazine systems with synthetic sphingolipid 2-hydroxymethyl-3-aryl octyl pyrrolidine. Interestingly, the pyrrolidine derivative (2*S*,3*R*)-3-(4-octylphenyl)pyrrolidin-2-yl)methanol exhibited cytotoxic activity against murine hematopoietic FL5.12 cells that have a metabolism similar to cancer cells when supplied with high levels of cytokine IL-3, which inhibited nutrient transporters, and lead to cell death [52]. Possibly, by the combination of this and similar compounds and the phenothiazine system, synergistic cytotoxic activity in cancer cells can occur. In this context, it is known that perphenazine, a phenothiazine drug, activated a key regulator of tumor cell apoptosis, which causes autophagy involving the protein phosphatase 2A (PP2A) [53]. However, the cytotoxicity was reduced when phenoxazine or phenothiazine were linked with (2*S*,3*R*)-3-(4-octylphenyl)pyrrolidin-2-yl)methanol, suggesting that combining the structural elements of multiple PP2A activators to achieve synergistic cytotoxicity was not a viable strategy, as exemplified by the only phenothiazine hybrid developed (compound **33**, Figure 19) [52]. However, this hybrid presents some nutrient transporter down-regulation at 40 µM (29% CD98; vacuolation score = 42), which was modest when compared to sphingolipid ((2*S*,3*R*)-3-(4-octylphenyl)pyrrolidin-2-yl)methanol (compound **33**) at 2.5 µM (32% CD98; vacuolation score= 46) [52].

## 3. Anti-Alzheimer Hybrids

**Figure 20:**
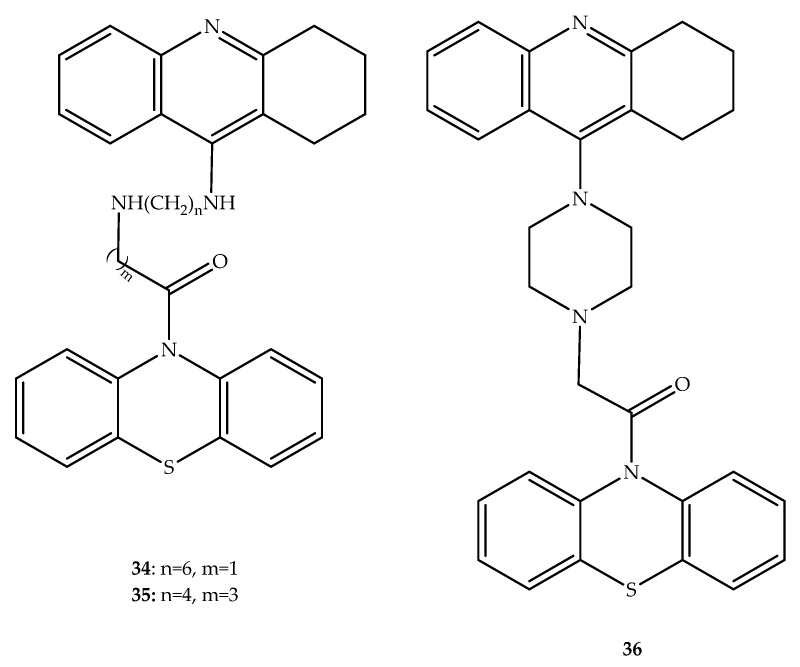
Chemical structure of the three most potent tacrine-phenothiazine hybrids with interest in Alzheimer’s disease.

Alzheimer’s disease is a progressive neurodegenerative disorder with a high societal impact. Many hypotheses regarding its pathogenesis have been considered, with the cholinergic and amyloid hypotheses being the most common. In fact, neurological deficits have been associated with decreases in acetylcholine, whose levels are regulated by AChE and butyrylcholinesterase (BuChE) enzymes. Therefore, inhibitors of these enzymes can prevent acetylcholine destruction in synapses [54]. In addition, there are two types of neuropathological changes in this disorder: extracellular β-amyloid plaques (Aβ) and intracellular neurofibrillary tangles [55]. Nowadays, the treatment of Alzheimer’s disease is only symptomatic and involves the use of cholinesterase inhibitors and memantine, a dopamine agonist and antagonist of the *N*-methyl-*D*-aspartate receptor. This treatment can be used in combination with other therapies, namely, to treat agitation and aggression symptoms [56]. Therefore, disease-modifying therapies that target amyloid cascade and Tau aggregates are highly necessary and have to be developed. Tacrine, an anti-Alzheimer drug that acts as a potent inhibitor of AChE and BuChE enzymes, was combined with the phenothiazine core in the development of new therapeutic approaches. In this context, the use of a phenothiazine moiety can be interesting because methylene blue, the first synthesized compound bearing the phenothiazine skeleton, was described as a protein Tau aggregation inhibitor and is in clinical phase II for the treatment of mild to moderate Alzheimer’s disease [54]. In comparison with tacrine, these hybrids (compounds **34**, **35** and **36**) developed by Hui et al. (2014) were found to be more effective as protein Tau aggregation and AChE inhibitors. In fact, three hybrids (Figure 20) have relevant effects in inhibiting the P-Tau (26.4–39.5% inhibition at 10 µM) and AchE (IC_50_ range = 89–217 nM). These effects were superior to those observed with tacrine (10.6% P-Tau inhibition at 10 µM and IC_50_ of AchE inhibitor = 275 nM). In addition, the hybrid **34** was also able to bind Aβ fibril (fAβ) and could react with immobilized fAβ, which may indicate effects in different fAβ assembly states [57,58].

**Figure 21:**
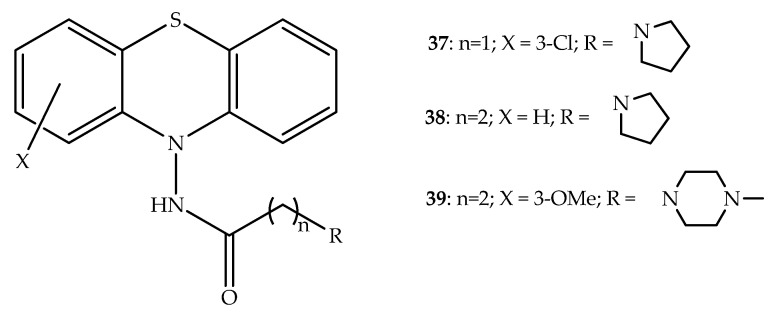
General chemical structure of *N*-acylaminophenothiazine hybrids with interest in neurodegenerative diseases.

A group of new multifunctional compounds with neuroprotective and anticholinergic properties was developed. In fact, these hybrids were found to have selective inhibitory activity against equine BuChE, but not against AChE. The new compounds with a hydrogen or chlorine atom at position 3 of the phenothiazine ring also demonstrated a similar effect. Three hybrid compounds (**37**, **38** and **39**; Figure 21) also demonstrated neuroprotection in neuroblastoma cells [59]. Based on findings from previous reports of the same research group, it was determined that the acylamino moiety was able to hybridize with phenothiazine and that the resulting hybrids would have antioxidant activity by sequestration of exogenous and mitochondrial free radicals, and BuChE inhibitory activity. The second mechanism appears to be linked with an elevation of the calcium levels in L-type calcium channels, and thus these compounds have multifunctional activity [59,60]. Therefore, these molecules may be useful to develop new drugs for the treatment of Alzheimer’s disease [59].

**Figure 22:**
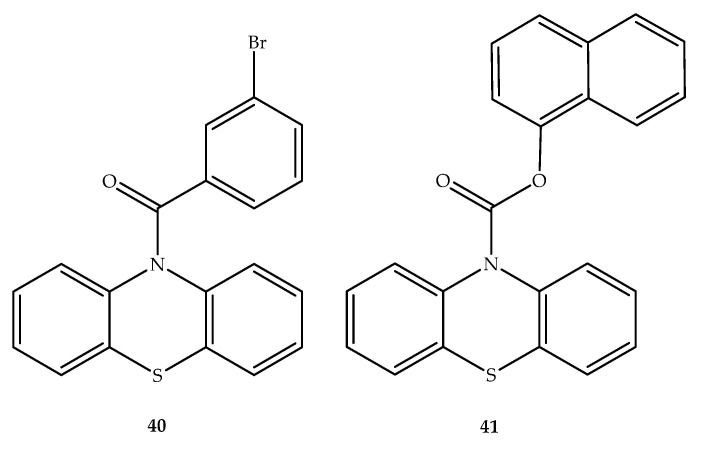
Chemical structures of *N*-carbonyl compounds butyrylcholinesterase inhibitors with no neurotransmitter receptor interactions.

Several carbonyl derivatives of phenothiazine were designed involving the replacement of the hydrogen linked to the phenothiazine nitrogen atom to originate different *N*-10 carbonyl amides and carbamates with potential interest in the treatment of Alzheimer’s disease. As classical antipsychotic phenothiazines have effects on several neurotransmitter receptors, in addition to AChE and BuChE inhibitory effects, the interference of these carbonyl derivatives of phenothiazine in different neurotransmitter receptors and transporters were also evaluated with an aim to develop compounds with undesirable effects. Among all the hybrids studied, compounds **40** and **41** (Figure 22) were selected as potent and reversible BuChE inhibitors, given that they interact poorly with dopamine transporter proteins and have a limited interaction with different CNS receptors. Therefore, these compounds were presented by the authors of this study as potential new Alzheimer’s disease therapeutics [61].

## 4. Antihistaminic Hybrids

**Figure 23:**
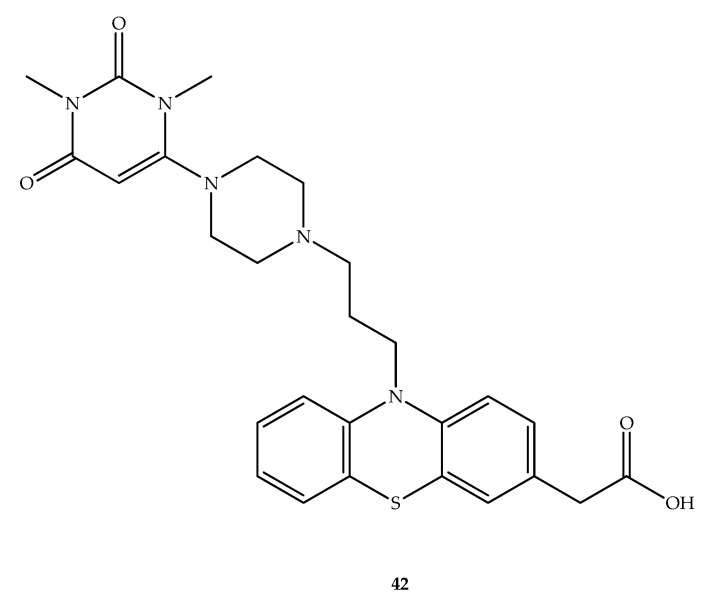
Chemical structure of hybrid **42** designed by Kubota et al. (2009) with antihistaminic activity [63].

Four subtypes of histamine receptors play a role in diverse physiological and pathophysiological processes in the body. Probably, the most important of these are H_1_ receptors, which are present in several types of cells, including inflammatory and immune cells. H_1_ receptor antagonists have been mainly clinically used to treat allergic-related conditions [62]. In this context, hybrids combining the 6-amino-pyrimidine-2,4(1*H*,3*H*)-dione moiety with phenothiazine carboxylic acid derivatives with diverse linkers were developed. Within this group, compound **42** (Figure 23) was selected as it inhibited both immediate (73%) and late (64%)-type allergic reactions at 10 mg/kg in the ovalbumine-induced biphasic cutaneous reaction mice model while exhibiting 61% in vivo inhibition of histamine induced skin vascular permeability at 3 mg/kg [63].

## 5. Antimicrobial Hybrids

### 5.1. Antibacterial Hybrids

**Figure 24:**
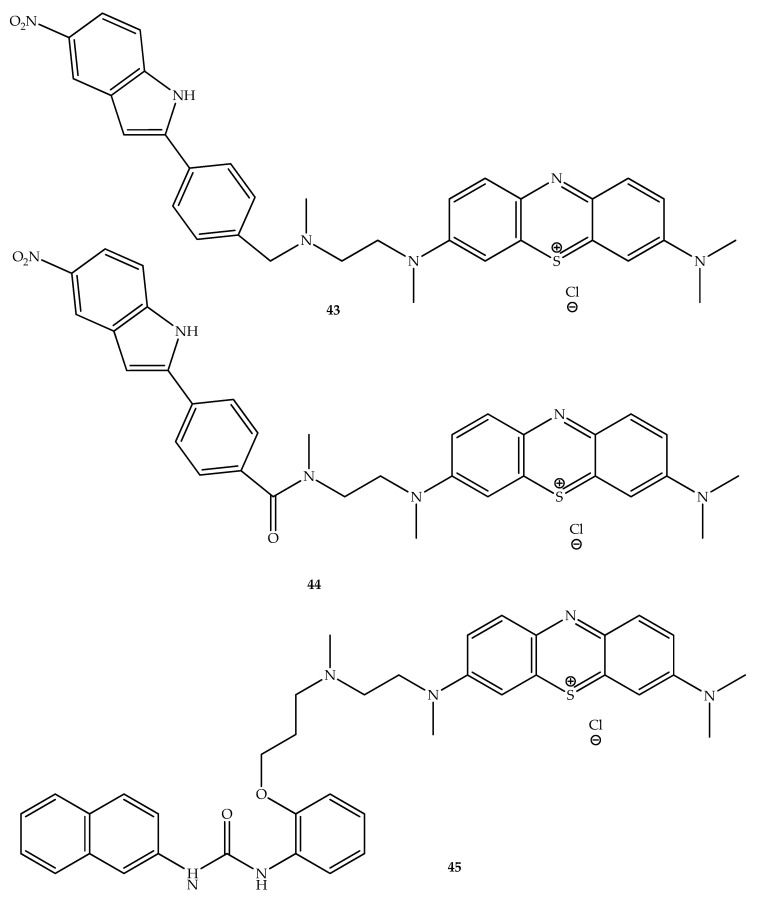
Chemical structure of phenothiazine hybrids with EPIs INF55 (**43** and **44**) and INF271 (**45**).

Despite recent efforts to curb drug resistance mechanisms, the effectiveness of current drugs continues to decline [64]. A potential strategy for restoring antibiotic activity may be to inhibit efflux pumps, which may confer multidrug resistance to bacteria. In this ambit, phenothiazines possess a wide range of properties including inhibition of antibiotic extrusion by targeting and inhibiting efflux pumps [65]. In fact, chlorpromazine exhibits efflux inhibitory activity by acting as a substrate for AcrB from the AcrAB-TolC complex of the resistance nodulation division family [66]. Therefore, combining phenothiazine cores with other antibacterial molecules can create an antibacterial drug that potentially overcomes bacterial drug resistance. In addition, for treating infections caused by microorganism that overexpress efflux pumps, phenothiazine systems can be hybridized with other efflux pump inhibitors (EPIs). Cationic phenothiazinium dyes can be used as antimicrobial agents in photodynamic therapy, and can be combined with an EPI to maximize photodynamic inactivation [67,68]. Interestingly, hybrid compounds containing NorA efflux pump inhibitors and methylene blue, a phenothiazinium dye, were shown to enhance photodynamic inactivation of methicillin-resistant *Staphylococcus aureus* (MRSA) in comparison with isolated methylene blue. A hybrid consisting of methylene-blue linked to an indole with efflux pump inhibitory properties (INF55) (compound **44**, Figure 24) has interesting photodynamic antimicrobial activity in in vitro *S. aureus* cultures and in an in vivo murine model infected with MRSA, and is able to treat localized multi-drug-resistant MRSA infections acting as efflux pump inhibitor and presenting an improved photodynamic activity and wound healing effects [69]. In addition, methylene-blue linked with efflux pump inhibitors INF55 (**43** and **44**, Figure 24) and INF 271 (a biphenyl urea, compound **45**) hybrids reduced survival of *E. coli* and *Acinetobacter baumannii* as demonstrated by antibacterial photodynamic inactivation in vitro and in vivo compared with treatment in presence or absence of methylene blue [70].

**Figure 25:**
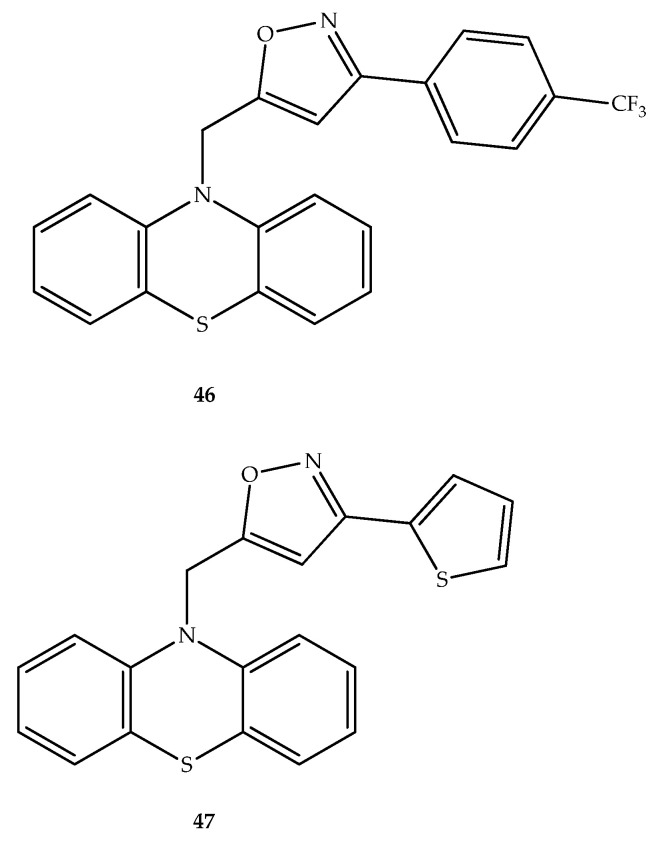
Chemical structure of isoxazole–phenothiazine hybrids with antibacterial effects.

In another work, a series of isoxazole-phenothiazine hybrids were also synthesized, and it was found an interesting antibacterial activity against Gram-Positive *Bacillus sphaericus* and *Staphylococcus epidermidis* and gram-negative *Klebsiella pneumonia* and *Escherichia coli*. This activity was higher or at least near to the observed activity with the antibacterial control (penicillin-G or streptomycin). In this group, compounds **46** and **47** (Figure 25) presented higher effects against all bacteria tested than the observed activity from antimicrobial penicillin-G (for Gram-positive bacteria) and streptomycin (for Gram-negative bacteria). Compound **46**, possessing a 4-trifluoromethyl group on the phenyl ring, also presents the best docking score in in silico simulations of the interaction with *Staphylococcus aureus* Murb, which express a homologous *E. coli* enzyme UDP-*N*-acetylenolpyruvylglucosamine reductase that is related with the synthesis of UDP-*N*-acetylmuramic acid in the bacterial cell wall [71,72].

**Figure 26:**
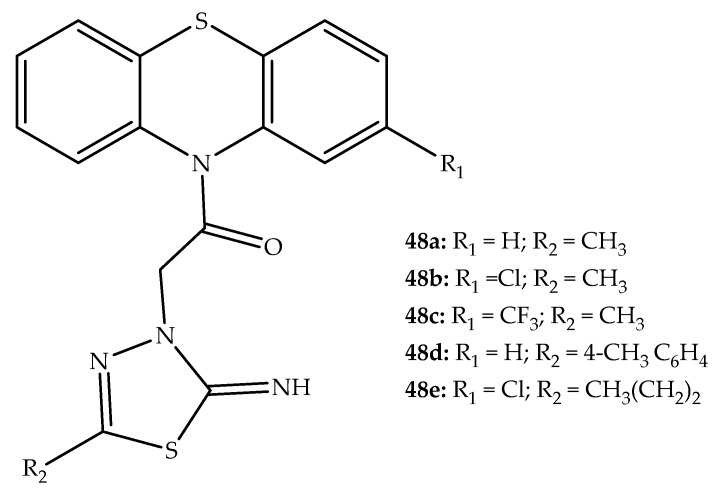
Chemical structure of the most potent phenothiazine-1,3,4-thiadiazoles developed by Ramprasad et al. (2015) [73].

In addition, relatively similar hybrids combining phenothiazine with 1,3,4-thiadiazole (Figure 26) showed good activity against gram-positive and gram-negative bacteria. One of the synthesized compounds (**48d**; Figure 26) was shown to be effective against *S. aureus*, *E. coli*, and *P. aeruginosa* in a disc diffusion assay. The potentiation of the inhibitory activity was associated with an alkyl or a substituted phenyl ring on the 1,3,4-thiadiazole ring. Several of these hybrids (**48a**, **48b**, **48c**, and **48d**) also presented relevant antitubercular activity (following section) [73].

### 5.2. Antitubercular Hybrids

The antitubercular phenothiazine hybrids that gained attention were discovered in the studies looking at hybrid antibacterial agents. The development of resistance to single drug therapy by *Mycobacterium tuberculosis* has driven the development of new therapeutic approaches to treat infection caused by this bacterium. In this context, it is possible to combine well-known antitubercular drugs with other scaffolds to act in different targets involved in the pathogenesis of tuberculosis and overcome the problem of resistance [74]. Interestingly, antitubercular phenothiazines that can act by inhibiting *M. tuberculosis* type II nicotinamide adenine dinucleotide dehydrogenases (NDH-2) were discovered [74]. This target is very advantageous since many pathogens produce it, but mammals do not. [75].

1,3,4-Thiadiazole-phenothiazine derivatives were tested against *M. tuberculosis* using assays similar to those performed for other bacteria. An evaluation of eight hybrids of this class revealed a minimum inhibitory concentration (MIC) of 1.6 µg/mL, comparable to standard drugs such as ethambutol and ciprofloxacin, which have MIC values of 3.125 µg/mL. Interestingly, a significant increase in antitubercular activity was observed when the R2 substituent in the 1,3,4-thiadiazole ring was a methylphenyl or an *n*-propyl group (Figure 26). In fact, two of the most potent synthetic hybrids (**48d** and **48e**, MIC = 0.8 µg/mL) possessed this substitution profile (Figure 26). Additionally, the most potent compounds had low toxicity profiles against VERO cells (IC_50_ > 215 µg/mL) and a selectivity index >10, which would be considered relatively non-toxic [73].

**Figure 27:**
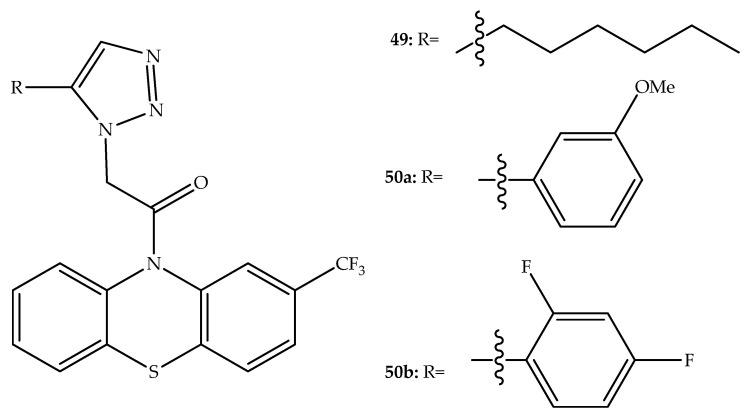
Chemical structure of phenothiazine triazole-based antitubercular agents.

Analogs of I-A09, a triazole-based antitubercular agent, were developed by combining a *N*-substituted 1,2,3-triazole and the tricyclic skeleton of trifluoperazine (Figure 27). Twenty-two of the synthesized compounds have antitubercular activity within a MIC range of 6.25 to 50 µg/mL. While all these compounds were less potent than isoniazid (MIC = 0.1 µg/mL) and ethambutol (MIC = 3.13 µg/mL), twenty were more active than pyrazinamide (MIC = 50 µg/mL). The most potent compounds contain an electron-donating methoxy group (**50a**) or two fluoro substituents (**50b**) on the phenyl ring, or a relatively long alkyl chain attached to the 1,2,3-triazole ring (**49**) (Figure 27). The cytotoxicity presented by these three hybrids against the embryonic kidney cell line (HEK-293T) demonstrated a high selectivity index. In fact, the most promising antitubercular agents (MIC = 6.25 μg/mL) presented HEK-293T cell growth inhibition in the range of 25.6–34.5% at 50 μg/mL [76].

**Figure 28:**
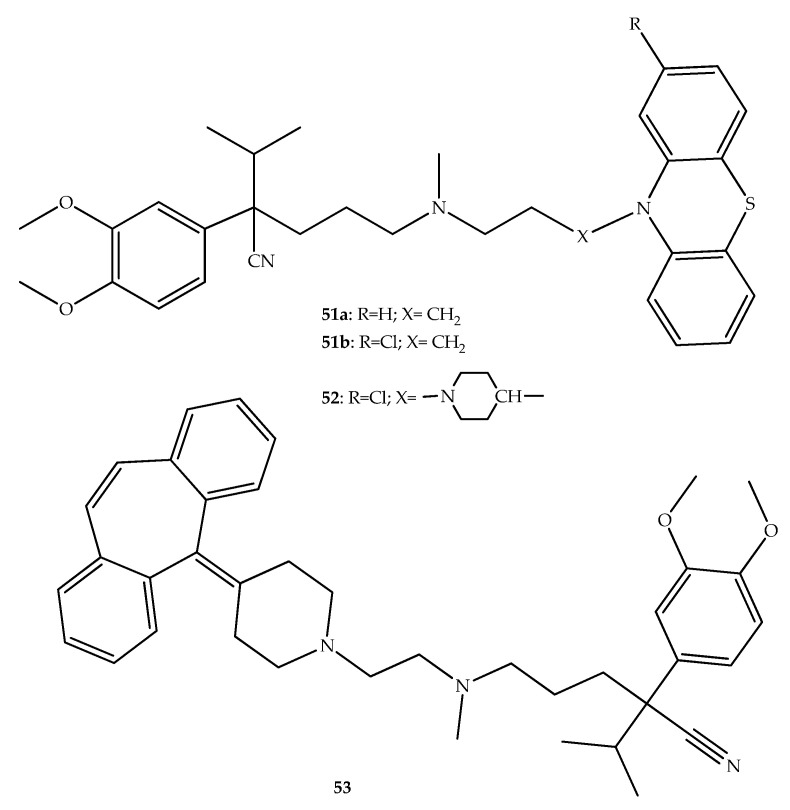
Chemical structure of verapamil phenothiazine hybrids with antitubercular interest.

In another work, Kumar et al. (2016) synthesized diverse EPI hybrids linking verapamil with phenothiazine or similar moieties (Figure 28) [77]. The cytotoxicity studies with compounds **51a** and **52** against THP-1 cells have shown IC_20_ values of 50–100 μg/mL, comparable with verapamil. The hybrids **51b** and **53** were found to be the least cytotoxic (IC_20_ > 100 μg/mL). When the activity of compounds **51a**, **51b**, **52**, and **53** (Figure 28) was tested, the hybrids **51a** and **51b** (MIC_90_ = 245.5 μg/mL and 128.9 μg/mL, respectively) showed poor intrinsic activity against *M. tuberculosis*, contrasting with **52** and **53** which showed relevant antitubercular activity (MIC_90_ values of 1.47 μg/mL and 3.17 μg/mL), respectively. Further, the combination of rifampicin and hybrids **51a**, **51b**, **52** and **53** was tested and a synergistic activity (fractional inhibitory concentration index (FICI) 0.4–0.5) was observed [77]. Moreover, compounds **52** and **53** were also shown to be capable of inhibiting efflux pump activity in intracellular *M. tuberculosis* on infected macrophages, which was observed as a potentiated effect when combined with rifampicin and isoniazid. Therefore, these compounds and combinations can be a viable strategy having the added advantage of being structures that possess intrinsic antitubercular activities and which may potentiate clinically used drugs to counteract resistance [77].

**Figure 29:**
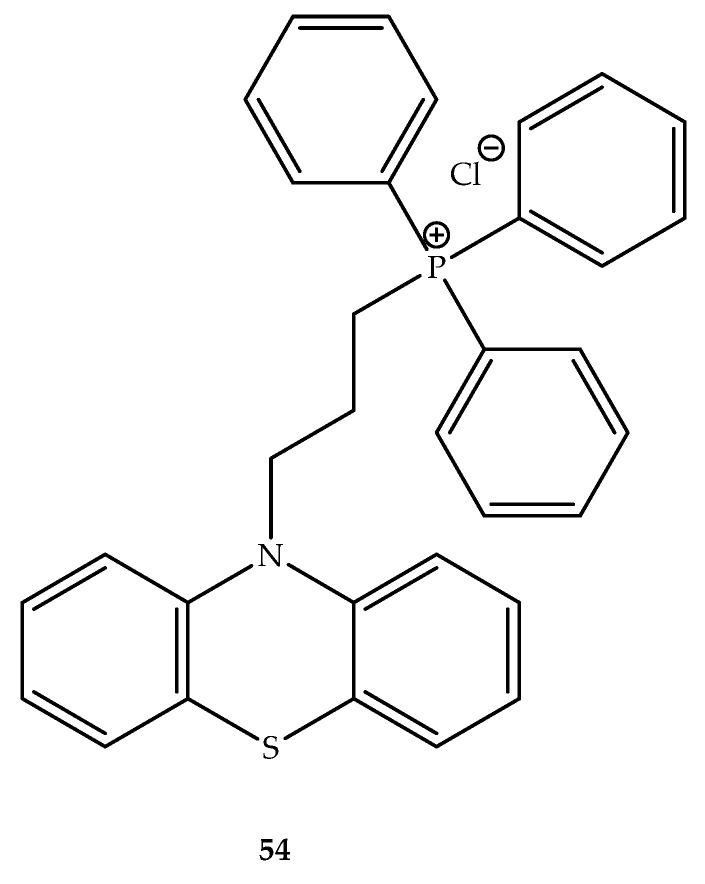
Chemical structure of a phenothiazines alkyltriphenylphosphonium hybrid (**54**) with activity against *M. tuberculosis*.

By coupling phenothiazines with alkyltriphenylphosphonium cations were obtained for compounds with improved localization and concentration at the mycobacterial membrane to target NDH-2. In this context, it was observed that the membrane accumulation in mycobacteria is related to compounds lipophilicity. In fact, the compounds synthesized that presented a specific lipophilicity (logP = 5.9–9.4) demonstrated an enhancement in the accumulation of *M. tuberculosis* and *Mycobacterium smegmatis*, and also in *S. aureus*. Of these synthesized compounds with this range of logP, compound **54** (Figure 29) inhibited the mycobacterial growth on a 0.5 µg/mL basis. A modification of the lipophilicity may enhance the specificity of phenothiazines to the mycobacteria membrane, which can reduce the pharmacological side effects associated with their action on the CNS. This can be achieved due to the difference between the membrane potential of *M. tuberculosis* (~160 mV) versus the CNS neurons (resting potential ~70 mV) [78].

**Figure 30:**
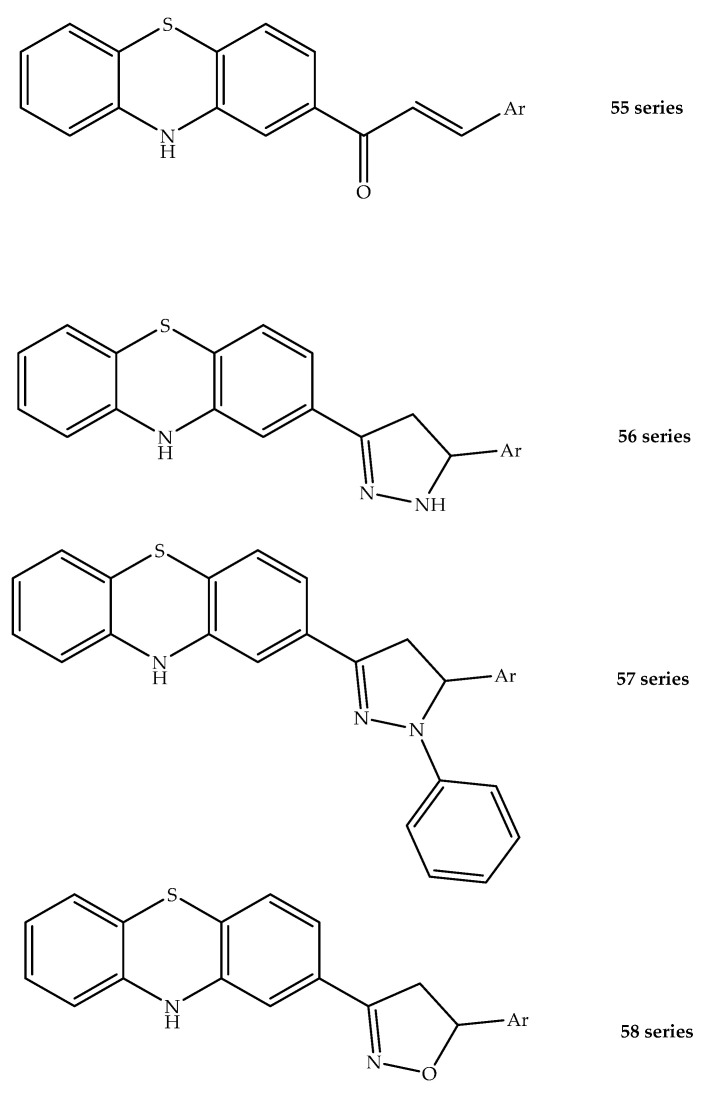
Chemical structures of phenothiazine linked with-chalcones, pyrazolines and isoxazolines demonstrating antitubercular activity.

Additionally, Alamar blue microplate assays were carried out to screen different phenothiazinylchalcones against *M. tuberculosis* H37Rv. Synthesized compounds from the phenothiazinylchalcone **55** series exhibited activity at 50 µg/mL and these compounds were used to prepare other series using different routes. Of these, the pyrazoline ring series **56** exhibited activity at 25 µg/mL. Adding a *N*-phenyl (**57** series) or the preparation of isoxazoline analogues (**58** series) (Figure 30) increased the potency of the hybrids, which had observed MICs of 12 and 6.25 µg/mL, respectively. According to an evaluation of the cytotoxicity against Vero cell lines and MICs against *M. tuberculosis*, the selectivity index of the compounds was determined, and the most active ones were considered safe and possessed selectivity indices higher than 10.25. Lipophilicity (logP > than 8.01) was also found to play a critical role in the antitubercular activity (MIC range 6.25–12.525 µg/mL) of these synthesized molecules [79].

**Figure 31:**
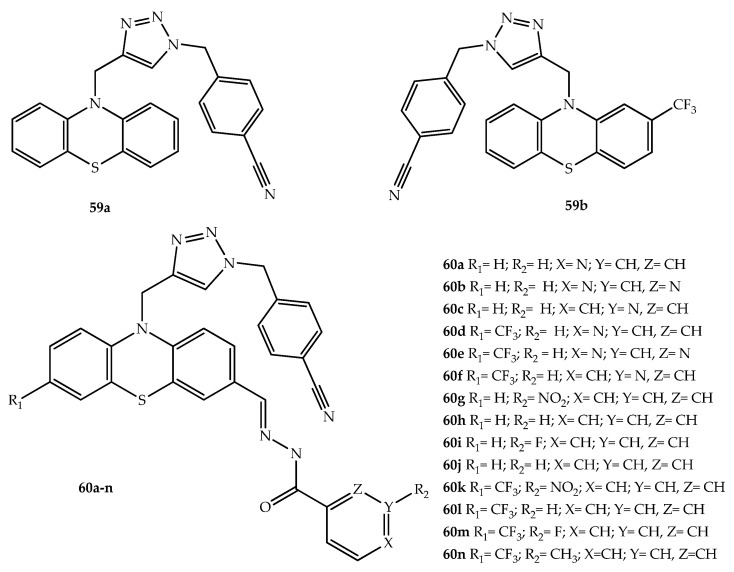
Chemical structures of phenothiazine-triazole hybrids with potent antitubercular activity.

Reddyrajula et al. (2019) designed hybrids with antitubercular activity by combining triazole and phenothiazine motifs by using distinct linkers [80]. Compared to compounds with ether or oxime linkers, phenothiazine-*N*-substituted 1,2,3-triazole derivatives demonstrated superior activity. Five compounds (**60b**, **60c**, **60e**, **60f** and **60h**) were two-fold more potent than pyrazinamide, an antibiotic used for the treatment of tuberculosis, in their antitubercular activity (MIC 1.6 µg/mL). A SAR study showed that electron withdrawing groups attached to the phenyl ring of 1,2,3-triazoles, such as NO_2_, OCH_3_, F and CN, are important factors for antitubercular and antimicrobial activity [80]. Compounds **59a** and **59b** (Figure 31) that were the two most potent molecules, were hybridized with various hydrazine derivatives to produce 14 compounds (**60a**–**n**; Figure 31) with similar antitubercular activity (MIC = 1.6 g/mL). The activity of some of these molecules was lower against *S. aureus*, *E. coli*, and *P. aeruginosa* than against *M. tuberculosis* thus indicating some selectivity. These compounds were nontoxic for Vero cells, with selectivity indices ranging from 42.25 to 796.99 at a concentration of 62.5 mg/mL [80]. An in silico adsorption, distribution, metabolism, and excretion, among another ten pharmacokinetic properties, assessment of these compounds showed potential good oral bioavailability, and molecular docking studies on Inh A and CYP121 enzymes showed their potential mechanisms of antitubercular activity. [80].

### 5.3. Antimalarial Hybrids

**Figure 32:**
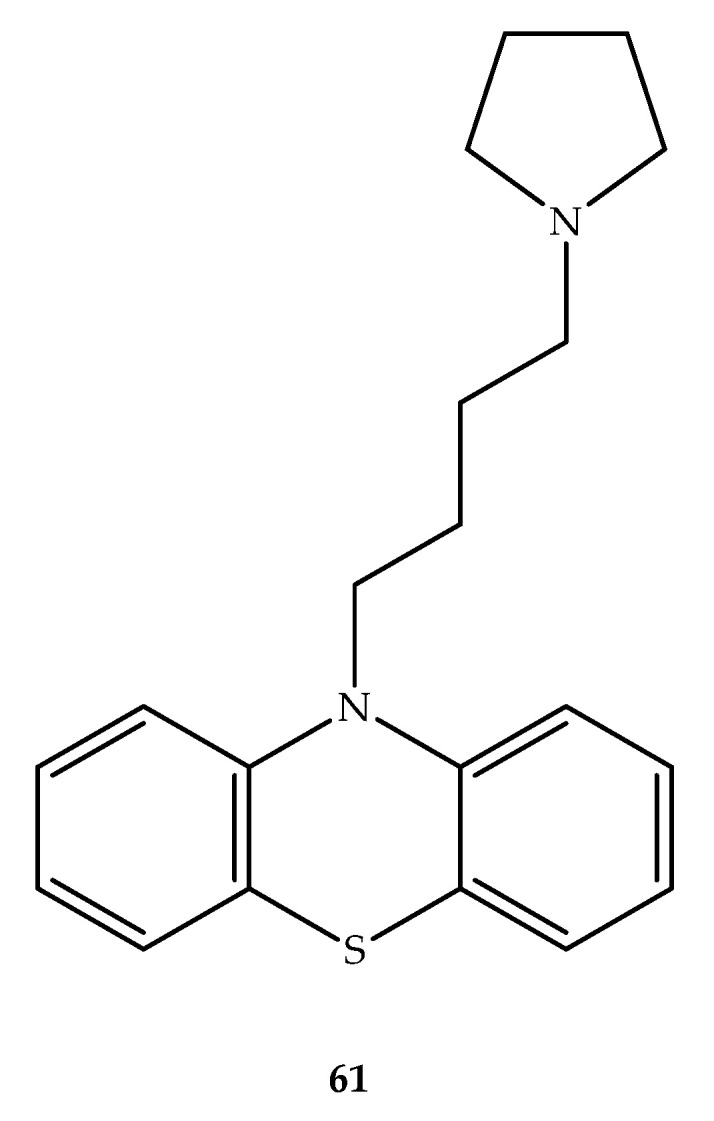
Chemical structure of compound **61**, an antimalarial that acts synergistically with chloroquine.

Unlike several other diseases, malaria is a multi-factorial condition in nature, meaning that both *Plasmodium* species parasites and the host are involved in determining the severity of the condition. Therefore, it is not surprising that several known single-targeting antimalarial agents, such as chloroquine and sulfa drugs can be ineffective in treating malaria and that resistances have largely been developed [81]. In this context, an effort was made to design a novel phenothiazine hybrid structure capable of modulating the resistance to drugs usually used to treat *P. falciparum* infections. These compounds were obtained by linking the phenothiazine system to a terminal amino group. The length of the chain linking the two cores ranged from four to six carbons and the terminal amine was cyclic or noncyclic. Interestingly, these compounds displayed moderate to good activity against resistant *P. falciparum*. The most effective compounds have chain lengths of four to six carbons and relatively simple terminal amine groups. In the evaluation of hybrids combination with chloroquine, compound **61** (Figure 32) showed a relevant synergistic modulatory activity (FICI = 0.21), which was more than twice as effective than chloroquine plus verapamil (FICI = 0.51), one of the best known chemosensitizers [82].

### 5.4. Antifungal Hybrids

**Figure 33:**
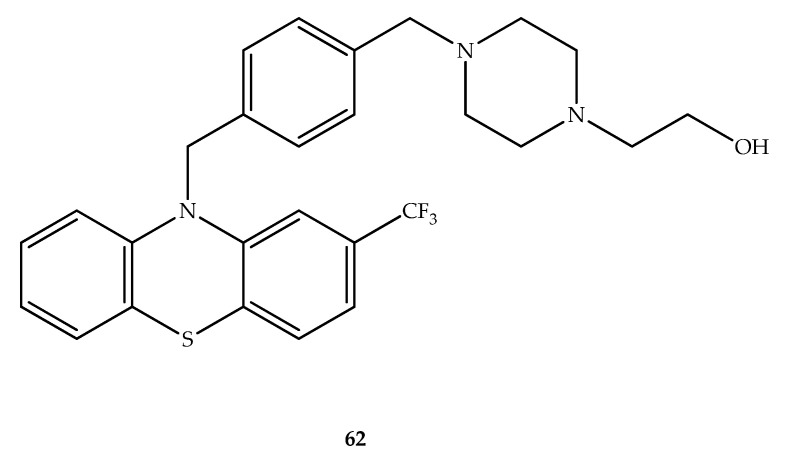
Chemical structure of compound **62** with antifungal activity.

Drug resistance to most antifungal drugs currently available is a major problem and thus the development of novel bioactive compounds in this context is highly needed. Antifungal activity has been demonstrated by some phenothiazines (e.g., chlorpromazine and promethazine) against planktonic cells and biofilms of *Cryptococcus* spp. [83]. Therefore, the discovery of new antifungal agents may benefit from the optimization of a phenothiazine scaffold. In this context, starting from conventional antipsychotic phenotiazines, several analogues with different structural variations were developed and studied as new anti-cryptococcal agents without marked CNS receptor binding affinities. Within the group of compounds studied those with amine linker chains longer than the existing ones in antipsychotic phenothiazines presented relevant anti-cryptococcal activity, with MIC values near 10 µg/mL. The most active derivative was compound **62** (Figure 33) (MIC against *C. neoformans* = 4 µg/mL), which also presented a good activity against *Candida albicans* (16 µg/mL) when compared with trifluoperazine (MIC > 32 µg/mL). It was further demonstrated that the hybrid **62** led to an additive inhibition with fluconazole (2-fold reduction) against *C. albicans* with mutations in its efflux pump, which makes the strain more resistant to fluconazole. Antagonism of calmodulin by the phenothiazine scaffold correlates well with its antifungal activity [84].

## 6. Anti-Obesity Hybrids

There is no doubt that obesity is a public health issue that leads to other conditions, such as type 2 diabetes and cancer, as well as cardiovascular and chronic kidney diseases. In this context, deregulated adipocyte physiology can lead to imbalanced energy storage and, consequently, to obesity and other diseases. Cannabinoid receptor type-1 (CB1) plays a critical role in controlling energy metabolism as well as stimulating adipogenesis and lipogenesis. In fact, when CB1 is specifically inhibited in adipocytes, a decrease in adipocyte size and in fat storage can occur and therefore CB1 antagonists can be useful in weight-reducing therapies and also have an appetite suppression effect [85]. In addition to obesity/metabolic disorders, the endocannabinoid system is also a therapeutic target for other diseases, including multiple sclerosis, anxiety and movement disorders [86].

**Figure 34:**
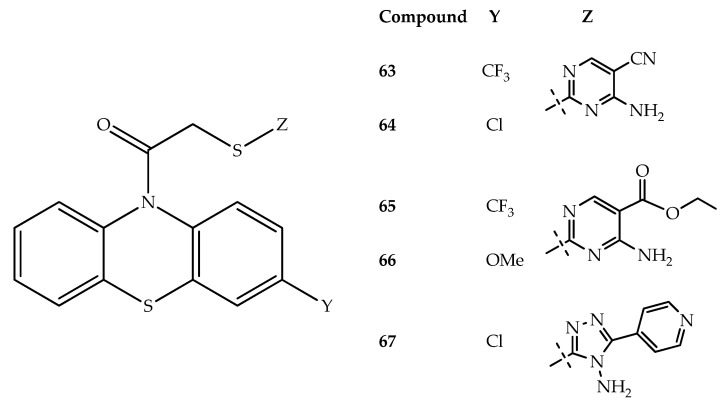
Chemical structure of the most active phenothiazine-CB1-antagonist hybrids.

A series of compounds presenting a phenothiazine scaffold was demonstrated to be potential CB1 receptor antagonists. Through three-dimensional quantitative SAR and molecular docking studies, the authors selected a hit molecule (**63**) that was chemically modified, and several biological tests were performed with the group of compounds prepared. Interestingly, four hybrids (**64**, **65**, **66** and **67**; Figure 34) were shown to reduce food intake in vivo [87]. The structure–activity relationship studies demonstrated that the replacement of the 4-aminopyrimidine-5-carbonitrile group (compound **63**) by an ethyl 4-aminopyrimidine-5-carboxylate (compound **65**) decreased the food intake (37%). The replacement of the trifluoromethyl group in compound **65** by a methoxy group (compound **66**) resulted in a 19% improvement in food intake. In addition, the change of the trifluoromethyl and 4-amino-pyrimidine-5-carbonitrile groups in **63** for chloro and 4-amino-5-(4-pyridyl)-4*H*-1,2,4-triazole groups, respectively, resulted in compound **67**, and this led to a 43% reduction in food intake. Further in vitro permeability studies with a PAMPA-BBB model demonstrated that the synthesized compounds should have low passive permeation to the CNS. This last result suggests a major action in the peripheral CB1 receptors in adipocytes, which may lead to anti-obesity activity with minimal side effects [87]. Chlorpromazine and thioridazine also demonstrated in vivo cannabinoid-like effects [88].

## 7. Analgesic and Anti-Inflammatory Properties

**Figure 35:**
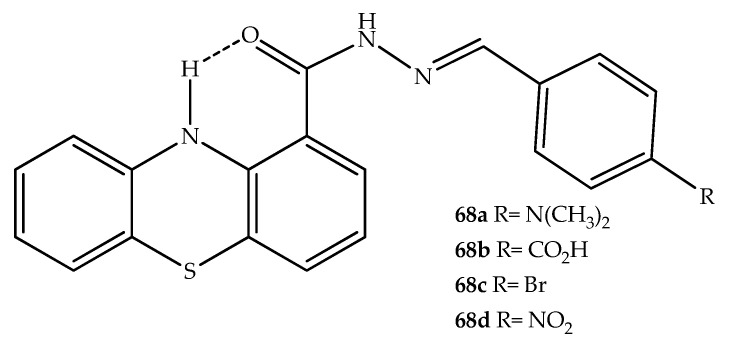
Chemical structures of compounds with antiplatelet and analgesic effects synthesized by Silva et al. (2004) [89].

Other medicinal properties of phenothiazine hybrids have been described. For example, 10*H*-phenothiazine-1-acylhydrazone hybrids (compounds **68a**–**d**; Figure 35) have been reported to exhibit analgesic properties, acting by inhibition of cyclooxygenase (COX) enzymes. In this context, two new analgesic compounds (**68b** and **68c**), which were more potent than dipyrone were developed. However, it was observed that there was some gastric irritability with compound **68b**, possibly because COX-1 was inhibited [89]. Interestingly, the new prototype compound (**68a**) also exhibited a potent antiplatelet effect. Thus, the inhibition of the COX-1-mediated formation of prostaglandins from arachidonic acid interfered in platelet aggregation [89].

Anti-inflammatory activity was also found in phenothiazine hybrids. The analysis of the protein’s denaturation was used to test novel phenothiazinyldihydropyridine dicarboxamides (**24a**–**h**; Figure 16). The drug used a standard was sodium diclofenac (90% denaturation). Interestingly, compounds **24b** and **24d**–**h** showed a high effect (near 100%) when compared with the standard drug. An increase in the anti-inflammatory activity was observed in molecules with more electronegative groups in the amide phenyl group [49]. Of these, compound **24d**, bearing a fluoro in the *ortho*-position, presented the most potent activity [49].

## 8. Conclusions

The pathophysiology of more complex diseases, such as cancer and Alzheimer’s diseases, usually involve many factors and therefore their treatments are frequently an enormous challenge. The use of a molecular hybridization approach allows the combination of two or more different pharmacophores, which makes available multiples mechanisms of action in a single molecule. The high diversity of the biological activities highlights the phenothiazine pharmacophore as a good candidate for molecular hybridization and, consequently, is being analyzed with this purpose. Therefore, the review presents for the first time, the most relevant phenothiazine hybrids with potential therapeutic interest. From the analysis of the joined information in this review, it is clear that a relevant group of new phenothiazine hybrids is being developed for the treatment of several diseases with high societal impact, such as cancer, microbial infections, Alzheimer’s disease and obesity. Interestingly, in several of these developed hybrids, the phenothiazine core was introduced due to its capacity to revert drug resistance in the treatment of cancer disease or different infections. In our opinion, as several of these compounds seem to be potent and selective, they deserve additional and more focused studies to further explore their potential application in clinical therapy. In conclusion, due to the increasing need for new drugs for the treatment of complex diseases, it is evident that the hybridization approach, namely involving the use of a phenothiazine core, can allow the development of new, safer and more effective molecules than classical drugs. Therefore, in the near future, an increase in the number of potential multitarget drugs developed through this medicinal chemistry approach can be expected.
